# Risk factors for pregnancy-related pelvic girdle pain: a scoping review

**DOI:** 10.1186/s12884-020-03442-5

**Published:** 2020-11-27

**Authors:** Francesca Wuytack, Cecily Begley, Deirdre Daly

**Affiliations:** School of Nursing & Midwifery, Trinity College Dublin, 24 D’Olier Street, Dublin 2, Ireland

**Keywords:** Pelvic girdle pain, Risk factors, Pregnancy, Scoping review

## Abstract

**Background:**

Pregnancy-related Pelvic Girdle Pain (PPGP) is a common complaint. The aetiology remains unclear and reports on risk factors for PPGP provide conflicting accounts. The aim of this scoping review was to map the body of literature on risk factors for experiencing PPGP.

**Methods:**

We searched the databases PubMed, Embase, CINAHL, PsycINFO, MIDIRS, and ClinicalTrial.gov (3 August 2020). We selected studies with two reviewers independently. Observational studies assessing risk factors for PPGP were included. Studies examining specific diagnostic tests or interventions were excluded.

**Results:**

We identified 5090 records from databases and 1077 from ClinicalTrial.gov. Twenty-four records met the inclusion criteria. A total of 148 factors were examined of which only 14 factors were examined in more than one study. Factors that were positively associated with PPGP included a history of low back or pelvic girdle pain, being overweight/obese, already having a child, younger age, lower educational level, no pre-pregnancy exercise, physically demanding work, previous back trauma/disease, progestin-intrauterine device use, stress, depression and anxiety.

**Conclusions:**

A large number of factors have been examined as potential risk factors for PPGP, but there is a lack of repetition to be able to draw stronger conclusions and pool studies in systematic reviews. Factors that have been examined in more than five studies include age, body mass index, parity and smoking. We suggest a systematic review be conducted to assess the role of these factors further in the development of PPGP.

**Supplementary Information:**

The online version contains supplementary material available at 10.1186/s12884-020-03442-5.

## Background

Pelvic girdle pain has been described as pain experienced between the posterior iliac crest (inferior to L5) and the inferior gluteal folds, particularly near the sacroiliac joints, and pain may radiate in the posterior thigh and can occur in conjunction with or separate from pain in the symphysis [1] ^(pp797)^. Women commonly experience pelvic girdle pain during pregnancy, with reported prevalence of Pregnancy-related Pelvic Girdle Pain (PPGP) ranging from 23 to 65% depending on the study methods used [2–4]. Women with PPGP often have impaired mobility, with 7 to 12.5% having to use crutches or a wheelchair [5–7]. PPGP symptoms affect their ability to cope with everyday life for which they feel unprepared and which they feel is not acknowledged [8–10]. PPGP is also a leading cause of sick leave during pregnancy [11–13].

The aetiology of PPGP remains uncertain but hormonal and mechanical factors have been suggested. Regarding hormonal factors, the main focus has been on the hormone relaxin, which is thought to increase pelvic laxity, yet evidence from clinical studies examining the association between relaxin and PPGP is of low quality with inconsistent results [14]. Biomechanically, there is moderate evidence that PPGP is associated with altered motor control, kinetic and kinematic parameters [15]. Knowledge of risk factors for PPGP can guide the development of preventative and management strategies, and can then lead to development of predictive models [16]. Existing narrative reviews, guidelines, background sections of primary studies and patient information vary widely in what they report as risk factors for PPGP and often seem conflicting when compared [1, 17, 18]. In their paper on the clinical presentation of PPGP, Wu et al. [19] included a structured review on risk factors and interpreted evidence as strong, weak, conflicting, or no evidence based on the number of studies, but numerous studies have been published since then.

The high PPGP prevalence, the increase in published studies on PPGP and the conflicting information in narrative accounts presented a clear need for a scoping review to examine and provide a comprehensive overview of the literature on risk factors for PPGP. A scoping review is a form of knowledge synthesis that maps, summarises and synthesises the evidence on a certain topic [20]. Scoping reviews may also be conducted to determine the value and scope of a full systematic review [21]. Preliminary searches identified a potentially large number of risk factors examined. Hence, a scoping review was perfectly placed for a thorough exploration of the literature to inform future systematic reviews. The aim of this scoping review was thus to map the body of literature on risk factors for experiencing PPGP and to identify research gaps.

## Methods

We searched five electronic databases (3 August 2020) including PubMed, Embase, CINAHL, PsycINFO, and MIDIRS with no time filters used. We also searched ClinicalTrial.gov (3 August 2020) for relevant registered studies. Only studies reported in English were included, but no language filter was applied to assess publication bias. We inspected reference lists of included studies and contacted experts in the field. The full search strategy is presented in Additional file 1.

Two review authors (FW, DD) independently reviewed all citations by title and by abstract. Where there was a lack of consensus, citations were moved to full-text selection. Any disagreement at full-text selection was resolved by discussion, and if necessary, involving a third reviewer. The selection criteria are presented in Table 1. Data were extracted independently by FW and DD using a piloted review-specific data extraction sheet. Data extracted included the year and country of publication, study design, setting, inclusion and exclusion criteria, number and characteristics of participants, the exposure and outcome measure(s) and how these were assessed, the number of participants with/without a certain exposure and outcome, and the risk estimates. Consensus was sought and, in addition, 25% of data were rechecked. Findings were summarised narratively and we have provided an overview of all factors examined in the literature in Tables 3, 4, 5 and 6. To map the existing literature on risk factors for PPGP, findings were grouped by trimester of pregnancy (first, second, third or any trimester/trimester not specified), by type of factor (physical, psychological, socio-demographic), and by whether they were examined in more than one study or not.

**Table 1:** Scope of the scoping review

**Population**	
Women who were pregnant at any gestation.	
**Exposure**	
Any potential risk factor defined as any modifiable or non-modifiable parameter that may increase or decrease the likelihood of a women experiencing PPGP. This excluded specific clinical tests and interventions.	
**Outcome**	
Pregnancy-related Pelvic Girdle Pain (PPGP) defined as any pain reported during pregnancy between the posterior iliac crest and the inferior gluteal fold, that may radiate in the posterior thigh and can also occur in conjunction with/or separately in the symphysis [1]	
**Study design**	
Observational prospective and retrospective cohort studies. Cross-sectional studies were included if they reported data on any factors that were present prior to the study. Intervention studies, case studies/reports, reviews, studies that explored overall prognosis, developed prediction models and stratified medicine research [22] were excluded. Only studies published in English were included.	

## Results

**Fig. 1:**
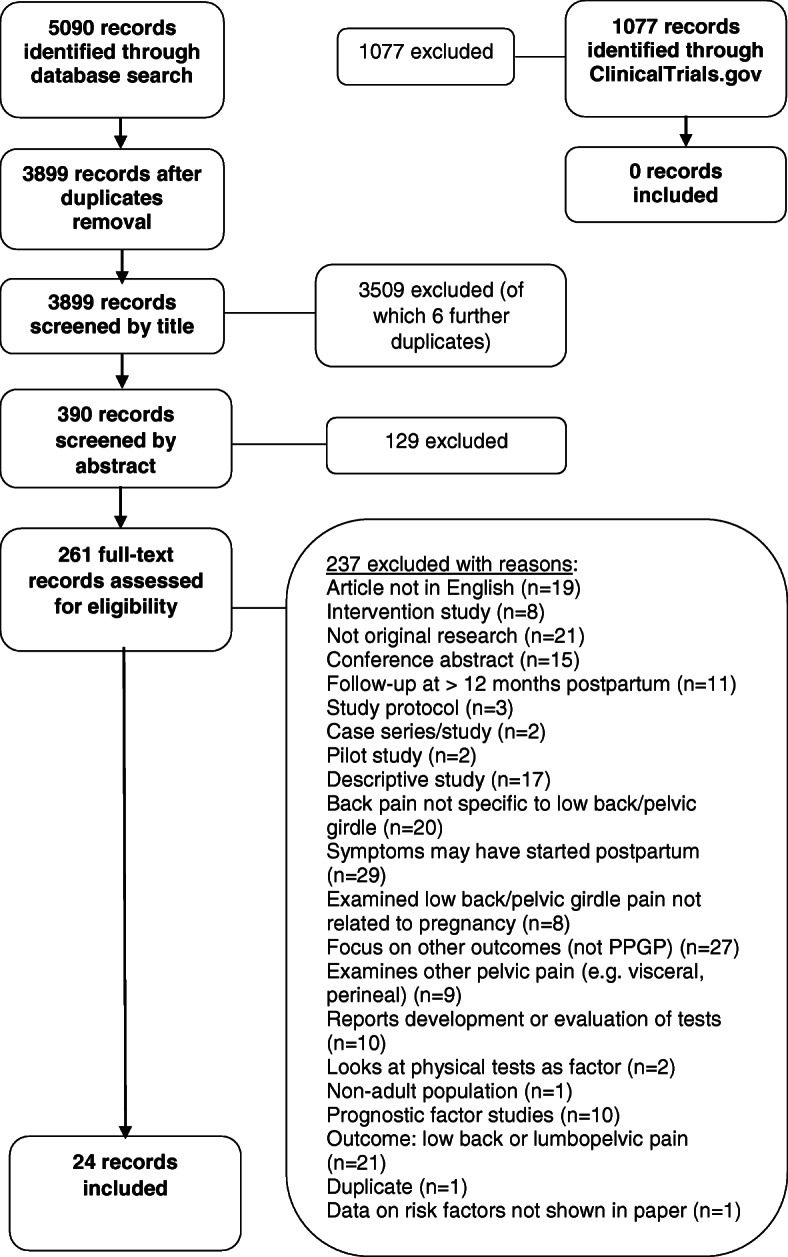
Flow chart of search results and study selection

We identified 5090 records from database searches. After duplicate removal, 3899 records were screened by title, 390 records by abstract, and 261 by full-text, of which 24 records met the inclusion criteria (Fig. 1). A total of 1077 records were identified from ClinicalTrials.gov one of which was eligible for inclusion but was a duplicate. Studies excluded at full text are listed by reason for exclusion in Additional file 5.

Five of the 24 included studies involved the same cohort, but because different risk factors were examined and reported in the publications, they were all included in the review [25, 28, 30, 31, 43]. The characteristics of the 24 included studies are presented in Table 2. Twelve prospective cohort studies, three retrospective studies and nine cross-sectional studies, published between 1995 and 2015 in Norway (*n* = 13), Sweden (*n* = 3), Denmark (*n* = 2), Japan (n = 1), Israel (n = 1), United Kingdom (n = 2), Brazil (n = 1) and Spain (n = 1), were included. All but one study [43] included both nulliparous and multiparous women, and only one study reported results separately for first-time mothers [28]. Eight of the 24 records examined the outcomes of low back pain or lumbopelvic pain during pregnancy in addition to PPGP outcomes, but only PPGP data were included as per review scope. Participants of 20 included studies completed self-reported questionnaires, three studies collected data from medical records [39, 42, 44] and one study conducted structured interviews with women [41]. Three studies also included a physical examination to assess for PPGP [24, 29, 40]. Seven studies examined the outcome Pelvic Girdle Syndrome (PGS), which is considered a subgroup of PPGP, defined as experiencing pain in the symphysis pubis and both sacro-iliac joints [24, 25, 28, 30, 31, 36, 43]. Seven studies examined pain at the anterior pelvic girdle as outcome, using the terms symphysiolysis [23, 41, 42], symphysis pubis dysfunction [44, 45] or pubis pain [34, 46]. Due to these differing inclusion criteria, methods of study, and stages of pregnancy or postpartum, few studies could be identified that would be suitable for meta-analysis in a future systematic review. As these occur, they are noted, and results on all other factors are presented in narrative format.

**Table 2:** Characteristics of included studies

Study (Country)	Study design^a^	Outcome(s)	Outcome assessment method	No nulliparous women	No of multiparous women	Risk factors examined
Albert et al 2006 [24] (Denmark)	P	PPGP; PGS; symphysiolysis; one-sided sacroiliac syndrome; double-sided sacroiliac syndrome	Self-reported questionnaire, and physical examination of ‘diseased group’ (=with PGP)	1103	1121	History of low back pain, trauma to the back, salpingitis previous year, multipara, oral contraceptive pill, hormone induced pregnancy, years since last pregnancy, weight before pregnancy, smoking, height, BMI, Social group 5 (no education), daily stress level, work satisfaction
Berg et al 1998 [23] (Sweden)	P	Symphysiolysis 20th week, symphysiolysis 30th week, symphysiolysis 35th week	Self-reported questionnaires in 20th, 30th and 35th week gestation	353	509	Parity, occupation
Bjelland et al 2010 [25] (Norway)	P	PGS^b^; Severe PGS^b^	Self-reported questionnaires (2nd trimester (mean 17.4 weeks, SD 2.8), 3rd trimester (mean 30.6 weeks SD 2))	35,084	40,871	Parity, maternal age, BMI, educational level, previous low back pain, emotional distress, physically demanding work, smoking, prepregnancy physical activity weekly
Bjelland et al 2011 [30] (Norway)	P	PGS^b^	Self-reported questionnaires (2nd trimester (mean 17.4 weeks, SD 2.8), 3rd trimester (mean 30.6 weeks SD 2))	34,676	42,297	Age of menarche
Bjelland et al 2013 [28] (Norway)	P	PGS^b^	Self-reported questionnaire (one in 2nd and 3rd trimester)	43,593	48,128	Combined oral contraceptive pills, progestin-only contraceptive pills, progestin injections, progestin intrauterine devices (in last year before pregnancy), combined oral contraceptive pills, progestin-only contraceptive pills, cessation of oral contraceptives (in 4 months before pregnancy and at time of being pregnant); Life-time duration of oral combined contraceptive pills, Life-time duration of progestin-only, contraceptive pills
Choratos et al 2015 (Norway)	P	PGS^b^	Self-reported questionnaire in week 15, weeks 18–22, and week 30 gestation.	27,492	24,156	Nausea, nausea and vomiting
Denison et al 2009 [44] (United Kingdom)	R	Symphysis pubis dysfunction	Retrospective analysis of antenatal notes and labour ward records.	Not stated (651 total)	Not stated (651 total)	BMI
Drevin et al 2015 [32] (Sweden)	C	Sacral pain	Drawing of the body to indicate any location of pain and time of onset of such pain. Indicated pain locations with onset before the present pregnancy were excluded.	Not stated (142 total)	Not stated (142 total)	Childhood physical abuse
Eberhard-Gran et al 2008 [36] (Norway)	C	PGS^b^	Self-reported questionnaire (sent to women with at least 1 prior delivery; any time after the delivery).	533	1283	Diabetes, BMI, time since last delivery, age at last delivery, parity
Endresen 1995 [33] (Norway)	C	PPGP^b^; PPGP^b^ that did not cause difficulties with housework; PPGP^b^ that caused difficulties with housework to some degree; PPGP^b^ that caused difficulties with housework to large/high degree; PLBP^b^; PPGP^b^ + Often PLBP^b^, PPGP^b^ + Rarely/Never PLBP^b^, PLBP^b^ + “yes” to PPGP^b^, PLBP^b^ + “No” to PPGP^b^	Self-administered questionnaire, completed postpartum while on the maternity ward.	2419	2746	Age, number of previous children, low back pain, parity, smoking, weight of newborn, work bending forward, woman’s year of birth, BMI, Strain at work, economic independence, twisting and bending, pelvic pain, education, work above shoulder, sex colleagues, frequent lifts 10-20 kg, permanently employed
Gjestland et al 2013 [26] (Norway)	P	PLPP^b^; PPGP^b^	Self-reported questionnaire (17–21 and 32 gestation, and 8 weeks and 2 years postpartum)	1700	1782	Exercise frequency
Hakansson et al 1994 [41] (Sweden)	P	Symphysiolysis	Structured interview	163	246	Manual work
Klemetti et al 2011 [45] (United Kingdom)	C	Symphysis pubis dysfunction	Self-reported questionnaire (completed postpartum)	Not stated (total 2825)	Not stated (total 2825)	Maternal age, parity
Kovacs et al 2012 [4] (Spain)	C	PLBP; PPGP	Self-reported questionnaire (28 weeks gestation or more) + clinical records	Not stated (1153 total)	Not stated (1153 total)	Smoking quantity, education level, work status, physical activity level, number of previous pregnancies, ≥1 previous instrumented delivery, ≥ 1 previous caesarean, ≥ 1 previous epidural anaesthesia, history of low back pain during previous pregnancy, history of low back pain not related to pregnancy, history of postpartum low back pain, experiencing low back pain around the time when getting pregnant, anxiety, depression, BMI, stage of pregnancy, Depression (BDI-II score), Anxiety (STAI score)
Kumle et al 2004 [37] (Norway)	C	PPGP^b^, PPGP in 1st pregnancy^b^ PPGP in 2nd pregnancy^b^, PPGP in 3rd pregnancy^b^	Self-reported questionnaire (retrospective questions any time after the birth)	307	1587	Hormonal contraceptive use before first birth, age of menarche, Age at first birth, time elapsed since first birth (per 3 years) (and enrolment in the cohort study), weight of newborn, years of education, smoking during first pregnancy, PPGP^b^ in first pregnancy, PPGP^b^ in at least one of the first 2 pregnancies, did not suffer from PPGP^b^ in the previous 2 pregnancies, PPGP^b^ in first but not in second pregnancy, PPGP^b^ not in first but in second pregnancy, PPGP^b^ both in first and in second pregnancy, hormonal contraceptives before first pregnancy, length of hormonal contraceptives before first pregnancy, progestin-only contraceptives, combined oestrogen progestin contraceptives
Larsen et al 1999 [40] (Denmark)	P	PPGP	Questionnaire at the routine prenatal examinations in week 16, 20, 30, 33, 38, and 40 (obstetrician or midwife) in which women were asked about pelvic pain. Plus, by a rheumatologist; interview, clinical and neurological examination performed to exclude any other cause of pelvic pain. Then interview by an occupational therapist and examination by a physiotherapist.	618	898	Being in work, heavy workloads to carry (> 10 kg), uncomfortable working positions, monotonous work, long walking distance at work, Working in draft and cold, working with chemicals, job satisfaction (positiviely speaking), working parttime, shiftwork, fixed salary, living in a house, more than three rooms in the house, lift at home, stairs more than 10 steps at home, living with or married to partner, children at home, doing more than 50% of the housework, symptom-giving PPGP in mother or sister, exercising regularly (once a week),smoking, primiparous, multiparous, pelvic pain in previous pregnancy, treatment for low back pain by doctor, treatment for low back pain by chiropractor, treatment for low back pain by physiotherapist, untreated low back pain, diseases in the back, bones, or joints, suffering from lower abdominal pain, other diseases, previous low back pain (while not pregnant), previous lower abdominal pain (while not pregnant), parity, weight, age
Lebel et al 2010 [42] (Israel)	R	Symphysiolysis	Reported by the women on admission or retrieved from her medical care files. Data were extracted from the computerized perinatal database of the hospital.	Not stated (80,898 total)	Not stated (80,898 total)	Previous caesarean section, recurrent abortion, mild pre-eclampsia, severe pre-eclampsia, chronic hypertension, diabetes mellitus (total), gestational diabetes mellitus, pregestational diabetes mellitus, premature rupture of membranes (PROM)
Malmqvist et al 2012 [35] (Norway)	R	Moderate to Severe PPGP	Self-reported questionnaire; retrospectively completed after the birth	219	350	Low back pain in previous pregnancies, pelvic girdle pain in previous pregnancies, low back pain in the year before pregnancy, pelvic girdle pain in the year before pregnancy, exercised at least 2–3 times a week before pregnancy, physically heavy work, primiparous, BMI before pregnancy, physical activity before pregnancy
Meucci et al 2020 [34] (Brazil)	C	Posterior PGP and Pubic symphysis pain	Self-reported questionnaire; Venn diagram	1156	1535	Age, smoking, diabetes, depression during pregnancy, number of pregnancies
Morino et al 2014 [46] (Japan)	P	Hip joint or pubis pain in 2nd trimester, Hip joint or pubis pain in 3rd trimester	Self-reported questionnaires; during 2nd (mean 22.4, SD 2.1 weeks) and 3rd trimester (mean 33.7 weeks, SD 2.1 weeks).	Not stated (355 total)	Not stated (355 total)	BMI
Owe et al 2015 [27] (Norway)	P	PGS^b^	Self-reported questionnaires (2nd trimester (mean 17.4 weeks, SD 2.2), 3rd trimester (mean 30.5 weeks SD 1.4))	39,184	0	Exercise frequency, exercise type
Robinson et al 2010 [3, 29] (Norway)	P	PPGP (Disability and pain intensity)	Self-reported questionnaires and clinical examination (blind to the questionnaire data) at time. Follow up in late pregnancy (30 weeks +) with questionnaire.	157	111	Pain location, disability rating index in early pregnancy, pain intensity in early pregnancy
Vangen et al 1999 [39] (Norway)	C	PPGP^b^	Hospital records	42	95	Pakistani, Norwegian
Wergeland & Strand 1998 [38] (Norway)	C	Disabling posterior pelvic pain (Posterior PPGP^b^); PLBP^b^	Self-reported questionnaire postpartum while still in hospital.	1615	1706	Influence on breaks at work, influence on work pace, externally paced work, manual work, lifting heavy loads (10-20 kg), influence on work content, Work with video display terminals, weekly hours of paid work ≥35, Weekly hours of paid work > 40, age, parity, education, partner education, daily smoking, coffee ≤4 cups

^a^*P* Prospective cohort study, *R* Retrospective cohort study, *C* Cross-sectional study, ^b^Questions to ascertain the outcome could be open to interpretation

### Risk factors for PPGP examined in more than one study

Eleven physical and three socio-demographic potential risk factors were examined in more than one study (Table 3). Findings are also provided in extended tabular format in additional file 2.

**Table 3:** Risk factor for PPGP examined in more than one study

Physical factors (number of studies (n))	Socio-demographic factors (number of studies (n))	Psychological factors
History of low back pain (n = 2)	Age (*n* = 7)	None examined
History of low back pain not related to pregnancy (*n* = 2)	Educational level (n = 4)	
Low back pain in previous pregnancies (n = 2)	Work satisfaction (n = 2)	
Pelvic girdle pain in previous pregnancies (n = 2)		
Age of menarche (n = 2)		
Parity (n = 9)		
Smoking (n = 8)		
Body Mass Index (n = 8)		
Maternal weight before pregnancy (n = 3)		
Maternal height (n = 2)		
Gestational diabetes (n = 2)		

A history of low back pain was a risk factor for PGS [24, 25] and for PPGP [4, 40]. Low back pain [4, 35] and pelvic girdle pain [35, 40] in previous pregnancies were also positively associated with PPGP. Women with an older age of menarche were at increased risk of PGS [30] and PPGP [37]. Nine studies examined parity as risk factor [23–25, 33, 35, 36, 38, 40, 45] and all but two studies [36, 40] found that parous women were more likely to have PPGP. Eight studies examined the relationship between smoking and PPGP and findings were conflicting with most studies finding no statistically significant association [4, 24, 25, 34, 37, 40]. Eight studies examined Body Mass Index (BMI) as a risk factor, with conflicting results. The relationship between pre-pregnancy weight and PPGP was conflicting but it was positively associated with PGS (*n* = 1880; AOR 1.03; *p* < 0.05) and symphysiolysis (“pain at the symphysis pubis”) (*n* = 1771; AOR 1.04; p < 0.05) [24]; however, only three studies examined this factor [4, 24, 40]. Two studies (*n* = 2224; *n* = 1149) found that maternal height was not a risk factor (*p* > 0.05) [4, 24]. The evidence concerning the association between PPGP and the weight of the newborn was conflicting [33, 37].

For socio-demographic factors, age was examined in seven studies with differing results. Four studies examined educational level of which three found that women with no university qualification were more at risk of PPGP (*n* = 946; OR 1.3 [1.0–1.8]; *p* = 0.03) [4], posterior PPGP (*n* = 2439; OR 1.3 [1.0–1.8]; *p* = 0.04) [38], and PGS (*n* = 21,397; AOR 1.3 [1.1–1.4]; *p* < 0.001) [25]. However, Malmqvist et al. [35] found no association between the years of education and moderate/severe PPGP (*n* = 306; SMD 0.03 [− 0.2–0.3]). Higher work satisfaction (2 studies) was associated with a reduced risk of PGS (*n* = 1880; AOR 0.9; *p* < 0.05) or double-sided sacro-iliac syndrome (*n* = 1314; AOR 0.9; *p* < 0.01), but not with single-sided sacro-iliac syndrome (*n* = 1961; *p* > 0.05) or symphysiolysis (*n* = 1771; p > 0.05) [24]. Evidence on the association between gestational diabetes and PPGP was conflicting [34, 42].

### Risk factors for PPGP in the second trimester of pregnancy examined in one study

Berg et al. [23] examined two physical potential risk factors for symphysiolysis in the second trimester and found no association with heavy or very heavy physical workload whether this included lifting movements (*n* = 451; OR 1.3 [0.6–2.5]; *p* = 0.5) or not (*n* = 513; OR 1.1 [0.6–2.1]; *p* = 0.7) (Table 4).

**Table 4:** Risk factor for PPGP in the second trimester examined in only one study

Physical factors	Socio-demographic factors	Psychological factors
Heavy or very heavy physical workload [23]	None examined	None examined
Heavy or very heavy physical workload including lifting movement [23]		

### Risk factors for PPGP in the third trimester of pregnancy examined in one study

Forty-two physical factors, nine psychological factors and two socio-demographic potential risk factors for PPGP in the third trimester of pregnancy were examined in ten studies (Table 5). Findings are also provided in extended tabular format in additional file 3.

**Table 5:** Risk factor for PPGP in the third trimester examined in only one study

Physical factors	Socio-demographic factors	Psychological factors
History of postpartum low back pain [4]	Social group 5 (no vocational training or professional education education) [24]	Depression: slightly, moderately, seriously (vs not) [4]
Experiencing low back pain around the time when getting pregnant [4]	Work status: currently working vs not working [4]	Depression (BDI=II score)
Physically demanding work (yes vs no) [25]		Daily stress levels [24]
Exercise frequency: 1–2 times, ≥3 times per week during pregnancy (vs < 1 per week) [26]		Anxiety: Traces of anxiety (vs normal) [4]
Exercise frequency before pregnancy: 1–3 times/month, 1–2 times/week, 3–5 times/week, ≥6 times/week (vs never) [27]		Anxiety: Pathological anxiety (vs normal) [4]
Exercise type: Brisk walking, Non-weight bearing, Low-impact exercises, High-impact exercises, Horseback riding, Mixed exercises (vs never) [27]		State Anxiety (STAI-S) [4]
Hours of exercise per week before pregnancy [4]		Trait Anxiety (STAI-T) [4]
Hours of exercise per week during pregnancy [4]		Anxiety (STAI score) [4]
Physical activity level: minimally, moderately active, very active (vs sedentary) [4]		Emotional distress: yes (≥2) (vs no (< 2)) [25]
Pre-pregnancy physical activity: < 1 per week, 1–2 per week (vs ≥3 per week) [25]		
Stage of pregnancy (weeks) [4]		
Lifetime duration of oral contraceptive pills: Combined oral contraceptive pills < 1 year, 1–3 years, 4–6 years, 7–9 years, ≥ 10 years (vs never) [28]		
Lifetime duration of oral contraceptive pills: progestin-only contraceptive pills < 1 year, 1–3 years, 4–6 years, 7–9 years, ≥ 10 years (vs never) [28]		
Combined OCP in last year before pregnancy (vs no hormonal contraception) [28]		
Progestin-only contraceptive pills in last year before pregnancy (vs no hormonal contraception) [28]		
Progestin injection in last year before pregnancy (vs no hormonal contraception) [28]		
Progestin intrauterine devices in last year before pregnancy vs no hormonal contraception [28]		
Combined oral contraceptive pill 4 months before pregnancy (vs no hormonal contraception in last year) [28]		
Progestin-only contraceptive pill 4 months before pregnancy (vs no hormonal contraception in last year) [28]		
Cessation of oral contraceptives 4 months before pregnancy (vs no hormonal contraception in last year) [28]		
Combined oral contraceptive pill at the time of being pregnant (vs no hormonal contraception in last year) [28]		
Progestin-only contraceptive pill at the time of being pregnant (vs no hormonal contraception in last year) [28]		
Cessation of oral contraceptives at the time of being pregnant (vs no hormonal contraception in last year) [28]		
Weight increase during pregnancy [24]		
Pain location: pubic symphysis vs no pain [29]		
Pain location: posterior pain only vs no pain [29]		
Pain location: posterior and pubic symphysis pain vs no pain [29]		
≥1 previous instrumental birth [4]		
≥1 previous caesarean [4]		
≥1 previous epidural anaesthesia [4]		
Disability rating index in early pregnancy [29]		
Trauma to the back [24]		
Years since last pregnancy [24]		
Salpingitis previous year [24]		
Hormone induced pregnancy [24]		
Oral Contraceptive Pill [24]		
Number of previous pregnancies: 2, 3, 4, 5 (vs 1) [4]		
Current weight (3rd trimester of pregnancy) [4]		
Age of menarche < 11 years, 11 years, 12 years, 13 years, 14 years (vs ≥14 years) [30]		
Nausea (only) in early pregnancy [31]		
Nausea and vomiting in early pregnancy [31]		
Childhood physical abuse [32]		

A history of postpartum low back pain, was positively associated with PPGP in the third trimester (*n* = 1164; OR 2.0 [1.4–2.8]; *p* = 0.0002) [4]. Women who had physically demanding work (4-point likert scale) were more likely to have PGS (*n* = 68,872; AOR 1.4 [1.4–1.5]; *p* < 0.001) [25]. A heavy or very heavy physical workload (*n* = 513; OR 1.9 [1.1–3.3]; *p* < 0.05) and a heavy or very heavy physical workload including lifting movements (n = 513; OR 1.9 [1.2–3.0]; p < 0.05) were also associated with an increased risk of symphysiolysis [23]. Women with pubic symphysis pain reported higher pain (*n* = 268; AOR 35.5 [19.7–51.1]; *p* < 0.001) and disability (n = 268; AOR 11.8 [2.3–21.2]; *p* = 0.03) than women with posterior pain only, as did women with combined pubic symphysis and posterior PPGP (n = 268; AOR (pain) 16.5 [1.8–31.1]; p < 0.001) [29]. Kovacs et al. [4] found women who had had instrument-assisted birth (*n* = 1164; OR 1.9 [1.4–2.6]; *p* < 0.0001) or epidural in previous birth (n = 1164; OR 1.5 [1.2–2]; *p* = 0.004) to be more likely to have PPGP. Disability in early pregnancy was a predictor of PPGP-related disability in the third trimester (n = 268; AOR 0.5 [0.3–0.6]; p < 0.001) [29]. Albert et al. [24] found that a history of back trauma was positively associated (*n* = 2224; AOR 2.8; p < 0.001) with all subgroups of PPGP except symphysiolysis (*n* = 1771; *p* > 0.05).

High-impact exercise pre-pregnancy reduced the risk of PGS (*n* = 12,964; Adjusted Risk Ratio (ARR) 0.8 [0.7–0.9]; *p* = 0.0005) [43] and Kovacs et al. [4] found that women who engaged in moderate activity were less likely to have PPGP (*n* = 582; OR 0.7 [0.5–0.9]; *p* = 0.02). Gjestland et al. [26] also found that women who exercised less than once a week were more likely to have PPGP (*n* = 1575; AOR 0.8 [0.6–1.0]; *p* = 0.01) when compared to women who exercised three or more times per week; however, the relation between pre-pregnancy exercise frequency and risk of PGS appears to be non-linear with a frequency of 3–5 times/week most likely to reduce the risk of PGS (n = 17,349; ARR 0.8 [0.8–0.9]; *p* = 0.003) [43]. While hormonal contraception was not a risk factor [24, 28], progestin intrauterine device use in the year before pregnancy was associated with an increased risk of PGS (*n* = 34,457; AOR 1.2 [1.1–1.3]; *p* < 0.001) in multiparous women [28]. Women who experienced nausea (*n* = 4020; AOR 1.9 [1.8–2.1]; *p* < 0.0001) and/or vomiting (*n* = 3946; AOR 2.3 [2.1–2.4]; p < 0.0001) in early pregnancy were more at risk of PGS [31]. Weight increase during pregnancy, years since last pregnancy, salpingitis in the previous year, having had a hormone induced pregnancy (*n* = 2224; *p* > 0.05) [24], and number of previous pregnancies (*n* = 1158; p > 0.05) [4] were not associated with PPGP. Childhood physical abuse was a risk factor for sacral pain during pregnancy (*n* = 142; AOR 4.4 [1.7–11.4]; *p* = 0.002) [32].

For psychological factors, depression (n = 1158; β coefficient 0.07 [0.04–0.1]; *p* < 0.001) and anxiety (*n* = 1149; *p* < 0.01) were associated with an increased risk of PPGP in univariate analysis [4]. Women with higher daily stress levels were more likely to have PGS (n = 2224; AOR 1.1; p < 0.001) and one-sided sacroiliac syndrome (*n* = 1961; AOR 1.1; *p* < 0.05), but not symphysiolysis (*n* = 1771; p > 0.05) or double-sided sacroiliac syndrome (*n* = 1914; p > 0.05) [24]. Higher emotional distress was also linked to an increased risk of PGS (*n* = 41,070; AOR 2.0 [1.8–2.3]; *p* < 0.00001) [25].

For socio-demographic factors, being in work (not defined) was associated with a decreased risk of PPGP (*n* = 1139; OR 0.8 [0.6–1.0]; *p* = 0.03) [4], and having had no vocational or professional education led to an increased risk of one-sided sacroiliac syndrome (n = 1961; AOR 0.5; p < 0.05) but was not associated with any other PPGP sub-outcome [24].

### Risk factors for PPGP in any trimester of pregnancy/trimester not specified examined in one study

Ten studies examined 48 physical factors, 29 socio-demographic factors, and one psychological factor in relationship to PPGP in any trimester of pregnancy (Table 6). Findings are also provided in extended tabular format in additional file 4.

**Table 6:** Risk factor for PPGP in any trimester/trimester not specified examined in only one study

Physical factors	Socio-demographic factors	Psychological factors
Low back pain during pregnancy [33]	Woman’s year of birth [33]	Depression while pregnant [34]
Low back pain in the year before pregnancy [35]	Age at last delivery: ≥25 (vs < 25 years) [36]	
Pelvic girdle pain in the year before pregnancy [35]	Age at first birth: 21–25, ≥26 (vs ≤20 years) [37]	
PPGP in the first pregnancy [37]	Partner’s education level: primary or secondary 9–10 years, secondary 11–12 years (vs university) [38]	
PPGP in at least 1 of the 2 first pregnancy [37]	Years of education: 10–12, 13–15,16+ (vs 7–9 years) [37]	
PPGP in the first 2 pregnancy [37]	Pakistani (vs Norwegian) [39]	
PPGP in the first but not 2nd pregnancy [37]	Being in work [40]	
PPGP in the 2nd but not the first pregnancy [37]	Monotonous work [40]	
Symptom-giving pelvic girdle relaxation in mother or sister [40]	Working part-time [40]	
Exercised at least 2–3 times/week before pregnancy [35]	Shiftwork [40]	
Pre-pregnancy physical activity [35]	Fixed salary [40]	
Regular exercise (once a week) [40]	Living in a house (yes vs no) [40]	
Combined OCP [37]	Having more than 3 rooms at home [40]	
Hormonal contraceptive use before first birth [37]	Having a lift at home [40]	
Length of hormonal contraceptive use 1–29 months, 30–59 months, 60 or more months (vs none) [37]	Having stairs with more than 10 steps at home [40]	
Progestin-only contraceptives [37]	Living with or married to partner [40]	
Diseases in the back, bones, or joints [40]	Children at home [40]	
Suffering from lower abdominal pain [40]	Doing more than 50% of the housework [40]	
Other diseases (other than diseases in the back, bones, or joints) [40]	Influence on breaks at work (yes vs no) [38]	
Previous lower abdominal pain (while not pregnant) [40]	Influence on work pace (yes vs no) [38]	
Lifting heavy loads at work (10-20 kg) [38]	Level of work pace control: No, low, medium (vs high) [38]	
Heavy loads to carry (> 10 kg) [40]	Externally paced work (yes vs no) [38]	
Physically heavy work [35]	Manual work (yes vs no) [38, 41]	
Strain at work (not clearly defined) [33]	Influence on work content (yes vs no) [38]	
Work bending forward [33]	Work with video display terminals (yes vs no) [38]	
Twisting and bending [33]	Weekly hours of paid work ≥35 (yes vs no) [38]	
Uncomfortable working positions [40]	Weekly hours of paid work > 40 (yes vs no) [38]	
Long walking distance at work [40]	Economic dependence [33]	
Stairs more than 10 steps at work [40]	Permanently employed [33]	
Working in draft and cold [40]		
Working with chemicals [40]		
Previous caesarean section [42]		
Recurrent abortion [42]		
Mild pre-eclampsia [42]		
Severe pre-eclampsia [42]		
Chronic hypertension [42]		
Diabetes mellitus [36, 42]		
Pregestational diabetes mellitus		
Premature rupture of membranes [42]		
Time since last delivery: < 5 years (vs 5 or more) [36]		
Time since first birth [37]		
≥ 4 cups of coffee (per day) [38]		
Treatment of low back pain by doctor (vs untreated) [40]		
Treatment of low back pain by chiropractor (vs untreated) [40]		
Treatment of low back pain by physiotherapist (vs untreated) [40]		
Untreated low back pain [40]		
Number of pregnancies [34]

**Table 7:** Recommendation for future research

Definition & terminology	Use the term Pelvic Girdle Pain (PGP) and its definition as outlined in the European Guidelines [1]. Clearly specify at what point or over what period in pregnancy that PGP was examined.
Assessment	Use a pain diagram to localise participants’ symptoms. If feasible, include a physical examination to identify women with PGP. If not, recognise this as a limitation. Report the exact questions asked and the full assessment procedure in detail.
Analysis	Conduct descriptive, univariable and multivariable analysis. Adjust for a history of PGP or conduct subgroup analysis by this factor.
Reporting	Fully report the descriptive data (proportion with PGP by the risk factor examined), univariable and multivariable analyses to facilitate pooling of findings in a systematic review.

Low back pain during pregnancy (*n* = 2853; β coefficient 0.514; *p* < 0.001) or having a history of untreated low back pain (*n* = 1516; OR 1.5 [1.1–2.0]; *p* = 0.01) was positively associated with PPGP [33, 40], but having received past treatment for low back pain from a doctor (*n* = 869; OR 1.6 [1.0–2.8]; *p* = 0.07), chiropractor (*n* = 1009; OR 0.8 [0.6–1.1]; *p* = 0.2) or physiotherapist (*n* = 1163; OR 0.8 [0.6–1.1]; p = 0.2) was not associated with PPGP [40]. Low back pain (*n* = 306; RR 3.5 [1.7–6.8]; *p* = 0.0004) or pelvic girdle pain (n = 306; RR 4.7 [1.8–11.8]; *p* = 0.001) in the year before pregnancy were also positively associated with moderate to severe PPGP [35], and a history of back, bone or joint disease (*n* = 1516; OR 2.4 [1.6–3.5]; *p* < 0.0001) or previous lower abdominal pain (*n* = 1516; AOR 3.1 [1.9–5.15]; *p* < 0.01) were associated with PPGP [40]. Women who had PPGP in their first pregnancy were more likely to have PPGP in their second pregnancy (*n* = 682; RR 57.3 [14.5–81.2]) [37], but the time since the first birth was not associated with PPGP (*n* = 1861; AOR 0.9 [0.8–1.0]) [37]. Lifting heavy loads (*n* = 3284; OR 1.3 [1.0–1.7]; *p* = 0.04), having to carry heavy loads (*n* = 1516; OR 1.9 [1.4–2.6]; *p* < 0.0001), bending forward (*n* = 3062; β coefficient 0.05; *p* < 0.05), twisting and bending at work (n = 3062; β coefficient 0.039; p < 0.05), working in uncomfortable positions (n = 1516; AOR 1.7 [1.1–2.5]; p < 0.05), working in a draft/cold (n = 1516; AOR 2.1 [1.4–3.1]; *p* = 0.01), and combined oral contraceptive (*n* = 1684; AOR 1.7 [1.2–2.3]) all carried increased risk of PPGP [33, 37, 38, 47], but strain at work (n = 3062; β coefficient 0.045; *p* > 0.05), having more than 10 steps of stairs at work (n = 1516; OR 1.1 [0.8–1.4]; *p* = 0.6), or working with chemicals (*n* = 1516; 1.1 [0.7–1.6]; *p* = 0.7) did not carry greater risk [33, 40]. Women who drank four or more cups of coffee daily were also more likely to report PPGP (*n* = 3286; OR 1.8 [1.3–2.4]; *p* = 0.0001) [38], and mild pre-eclampsia (*n* = 81,142; OR 2.2 [1.2–4.0]; p = 0.01), diabetes mellitus (n = 81,142; OR 1.8 [1.1–3.0]; *p* = 0.02) and gestational diabetes (n = 81,142; OR 1.8 [1.0–3.2]; *p* = 0.03) were positively associated with symphysiolysis [42]. Previous caesarean section (n = 81,142; OR 0.8 [0.5–1.3]; *p* = 0.4), recurrent abortion (n = 81,142; OR 1.4 [0.8–2.5]; *p* = 0.3), severe pre-eclampsia (n = 81,142; OR 0.5 [0.07–3.9]; *p* = 0.5), chronic hypertension (n = 81,142; OR 1.1 [0.4–3.6]; *p* = 0.8), pregestational diabetes mellitus (n = 81,142; OR 1.6 [0.5–5.15]; *p* = 0.4) and premature rupture of membranes (n = 81,142; OR 1.0 [0.6–1.8]; *p* = 0.9) were not associated with symphysiolysis [42]. Women who exercised once a week (*n* = 1516; AOR 0.6 [0.4–0.9]; *p* < 0.01) [40] or exercised at least two to three times a week before pregnancy (*n* = 306; OR 0.6 [0.3–0.9]; p = 0.02) [35] were less likely to have PPGP. There was no association between the number of pregnancies and PPGP [34].

For socio-demographic factors, there was no association between PPGP and level of influence on work pace control (*n* = 3272; OR 0.9 [0.7–1.2]; *p* = 0.6), number of paid working hours (*n* = 3168; OR 0.7 [0.4–1]; *p* = 0.08), having a fixed salary (*n* = 1516; OR 1.1 [0.3–5.1]; p = 0.9), or whether jobs involved manual work (*n* = 3273; OR 1.1 [0.9–1.4]; p = 0.3), video display terminals (*n* = 3187; OR 0.8 [0.6–1.1]; *p* = 0.1), part-time work (*n* = 1516; OR 1.0 [0.7–1.4]; *p* = 1.0), shift work (*n* = 1516; OR 0.8 [0.5–1.2]; *p* = 0.2) or monotonous work (n = 1516; OR 1.2 [0.8–1.8]; p = 0.4) [38, 40, 41]. However, women who had more influence on breaks at work were less likely to report PPGP (n = 3272; OR 0.7 [0.5–0.9]; *p* = 0.002) [38] and women who were permanently employed (*n* = 1737; β coefficient 0.102; *p* < 0.05) or economically dependent (*n* = 3062; β coefficient 0.052; p < 0.05) were more likely to report PPGP [33]. Women who were from Pakistani background (compared to Norwegian women) were less likely to have PPGP (*n* = 137; OR 0.4 [0.2–0.8]) [39] and women who had children already at home were also more likely to have PPGP (n = 1516; OR 2.2 [1.6–3.1]; *p* < 0.0001), but having more than three rooms in the house (n = 1516; OR 1.4 [1.0–2.2]; *p* = 0.08), having stairs (n = 1516; OR 0.9 [0.6–1.1] p = 0.2) or a lift at home (n = 1516; OR 0.6 [0.3–1.3]; p = 0.2), living with a partner (n = 1516; OR 0.6 [0.4–1.0]; *p* = 0.07), or doing more than half of the housework (n = 1516; OR 1.2 [0.9–1.6]; *p* = 0.3) did not impact on PPGP [40]. Women whose partner had primary or secondary level education were more likely to have PPGP than women whose partner had a university qualification (*n* = 1822; OR 1.4 [1.1–1.9]; *p* = 0.02) [38], but the number of years of education women had was not associated with PPGP (*n* = 1861; 10–12 years AOR 1.0 [0.7–1.3]; 13–15 years AOR 0.9 [0.7–1.3]; ≥16 years AOR 1.1 [0.7–1.7] (versus 7–9 years)) [37].

Depression during pregnancy was associated with PPGP and pubic symphysis pain during pregnancy (Adjusted Risk Ratio 2.74 [1.38–5.44]; *p* = 0.004) [34].

## Discussion

This review represents a comprehensive overview of the available evidence on risk factors for PPGP. A very large number of factors, 148 in total, were examined in 24 studies, yet only 14 factors were examined in more than one study. Definitions of PPGP varied across studies and only three studies included a physical examination despite it being recommended to differentiate PPGP from low back pain [1]. There is an urgent need for consistency in PPGP measurement to allow pooling of data in future systematic reviews. We recommend adhering to the definition of PGP outlined in the European Guidelines and including a physical examination as per the guidelines [1].

Based on the findings of this scoping review, we recommend that systematic reviews are performed on factors that were examined in multiple studies, in particular the factors age, BMI, parity, and smoking, which have been examined in more than five studies. This scoping review can also provide a basis to design robust prospective observational studies to increase our understanding of the development of PPGP. Considering the susceptibility of observational research to bias, repetition of studies is required to draw strong conclusions. This is currently lacking for most of the risk factors examined in the literature, making pooling of multiple studies impossible. Issues to consider include the consistency of the association across studies, and, if present, the strength and timing of the association, and the dose-response relation where appropriate. Such research can provide strategies to improve management of this common condition. This is particularly important given the clinical implications of persistent PPGP, which causes new mothers considerable pain and difficulty, sometimes for up to two years postpartum [48, 49]. Other concerns in some existing studies that should, where possible, be avoided in future are: small sample sizes, a lack of robust multivariable analysis, and incomplete reporting (of the response rate, assessment procedure, the findings and analysis). Prospective registration of observational studies would also help address some of these issues. Recommendations for future research are summarised in Table 7.

### Limitations

We conducted a scoping review of the literature on risk factors for PPGP and thus did not conduct a risk of bias assessment. Ideally, this study will be followed by systematic reviews on specific, potential risk factors that have been examined in more than one study to include a risk of bias assessment before synthesising the literature.

## Conclusions

This scoping review presents an overview of current literature on risk factors for PPGP. A total of 148 factors were examined in the included studies, but issues of varying definitions and measurement methods, and a lack of replication were identified, which makes meta-analysis impossible. This review provides a basis to guide future systematic reviews and research on the development of PPGP. In conclusion, the literature on risk factors for PPGP is incomplete and statements regarding such risk factors should reflect current limitations and uncertainty.

## Supplementary Information

**Additional file 1.** Search strategy.

**Additional file 2.** Risk factors for PPGP examined in more than one study.

**Additional file 3.** Risk factors for PPGP in the third trimester of pregnancy.

**Additional file 4.** Risk factors for PPGP in any trimester of pregnancy/trimester not stated.

**Additional file 5.** Studies excluded at full text selection by reason for exclusion.

## Data Availability

This study was a scoping review, hence no data was generated.

## Abbreviations

BMI: Body Mass Index
CI: Confidence Intervals
OR: Odds Ratios
PGS: Pelvic Girdle Syndrome
PPGP: Pregnancy-related Pelvic Girdle Pain
RR: Risk ratio
SMD: Standardised Mean Difference
