# Supplementary material for: Risk factors for pregnancy-related pelvic girdle pain: a scoping review
Source: BMC Pregnancy Childbirth. 2020 Nov 27;20:739. doi: 10.1186/s12884-020-03442-5 (PMC7694360; doi:10.1186/s12884-020-03442-5)
Supplement: Supplementary file 1 — Additional file 1. Search strategy. [file 12884_2020_3442_MOESM1_ESM.docx]

| **Additional file 1: Search Strategy** | | | |
| --- | --- | --- | --- |
| **Database (Date)** | **Filters** | **Search** | **Number of citations** |
| PubMed  (4 Dec 2014) | None | (“low back pain”[Mesh] OR “pelvic pain”[Mesh] OR “sacroiliac joint”[Mesh] OR “pelvis”[Mesh] OR “pubic symphysis”[Mesh] OR “sacrum”[Mesh] OR pelvic OR pelvis OR sacroiliac OR “sacro iliac” OR sacral OR sacrum OR “pubic symphysis” OR “symphysis pubis” OR symphyseal OR lumbopelvic OR lumbar OR back) AND (“pain”[Mesh] OR pain OR instability OR insufficiency OR subluxation) AND (“pregnancy”[Mesh] OR “pregnancy complications”[Mesh] OR “postpartum period”[Mesh] OR “parturition”[Mesh] OR pregnancy OR “ante natal*” OR prenatal* OR antenatal* OR “pre natal*” OR “prenatal*” OR birth* OR childbirth OR perinatal* OR “peri natal*” OR postpartum OR “post partum” OR postnatal* OR “post natal*”) AND (“prognosis”[Mesh] OR “risk factors”[Mesh] OR prognos* OR risk* OR predict* OR persist*) | 1738 |
| CINAHL  (4 Dec 2014) | None | ((MH "Pelvis") OR (MH "Pelvic Pain") OR (MH "Sacroiliac Joint") OR (MH "Sacroiliac Joint Dysfunction") OR (MH "Pubic Symphysis") OR (MH "Low Back Pain") OR (MH "Sacrum") OR pelvic OR pelvis OR sacroiliac OR “sacro iliac” OR sacral OR sacrum OR “pubic symphysis” OR “symphysis pubis” OR symphyseal OR lumbopelvic OR lumbar OR back) AND ((MH "Pain") OR pain OR instability OR insufficiency OR subluxation) AND ((MH "Pregnancy") OR (MH "Childbirth") OR (MH "Vaginal Birth") OR (MH "Term Birth") OR (MH "Postnatal Period") OR pregnancy OR “ante natal*” OR antenatal* OR prenatal* OR “pre natal*” OR birth OR childbirth OR perinatal* OR “peri natal*” OR postpartum OR “post partum” OR postnatal* OR “post natal*”) AND ((MH "Prognosis") OR (MH "Risk Assessment") OR prognos* OR risk* OR predict* OR persist*) | 278 |
| Maternity and Infant Care-MIDIRS online  (4 Dec 2014) | None | (“low back pain”.de. or “low back pain”.mp. or “pelvic pain”.de. or “sacroiliac joint”.de. or “pubic symphysis”.de. or pelvic.mp. or pelvis.mp. or sacroiliac.mp. or “sacro iliac”.mp. or “pubic symphysis”.mp. or “symphysis pubis”.mp. or sacrum.mp. or sacral.mp. or symphyseal.mp. or lumbopelvic.mp. or back.mp.) and (pain.de. or pain.mp. or instability.mp. or insufficiency.mp. or subluxation.mp.) and ((pregnancy or “postnatal period”).de. or pregnancy.mp. or antenatal*.mp. or “ante natal*”.mp. or prenatal*.mp. or “pre natal*”.mp. or postnatal*.mp. or “post natal*”.mp. or birth.mp. or childbirth.mp. or postpartum.mp. or “post partum”.mp. or perinatal*.mp. or “peri natal*”.mp.) and (prognos* or risk* or predict* or persist*).mp | 184 |
| PsycINFO  (4 Dec 2014) | None | (DE "Back (Anatomy)" OR DE "Back Pain" OR pelvic OR pelvis OR sacroiliac OR “sacro iliac” OR sacral OR sacrum OR pubic symphysis OR symphysis pubis OR symphyseal OR lumbopelvic OR lumbar OR back) AND (DE "Pain" OR DE "Chronic Pain" OR DE "Myofascial Pain" OR DE "Pain Perception" OR pain OR instability OR insufficiency OR subluxation) AND (DE "Pregnancy" OR DE "Prenatal Exposure" OR DE "Prenatal Care" OR DE "Perinatal Period" OR DE "Labor (Childbirth)" OR DE "Birth" OR DE "Postnatal Period" OR DE "Pregnancy Outcomes" OR DE "Birth Injuries" OR DE "Birth Trauma" OR DE "Obstetrical Complications" OR pregnancy OR “ante natal*” OR antenatal* OR prenatal OR “pre natal*” OR birth OR childbirth OR perinatal* OR “peri natal*” OR postpartum OR “post partum” OR postnatal* OR “post natal*”) AND (DE "Prognosis" OR DE "Disease Course" OR DE "Prediction" OR DE "Protective Factors" OR DE "Risk Assessment" OR DE "At Risk Populations" OR DE "Risk Factors" OR DE "Persistence" OR prognos* OR risk* OR predict* OR persist*) | 62 |
| EMBASE  (4 Dec 2014) | (without MEDLINE) | (('low back' OR 'pelvic girdle' OR 'sacroiliac joint' OR 'sacroiliac joints' OR pelvis OR pelvic OR lumbar OR pelvic OR sacroiliac OR 'sacro iliac' OR sacral OR sacrum OR coccyx OR coccygeal OR 'symphysis pubis' OR 'pubic symphysis' OR symphyseal OR lumbopelvic OR back) AND (pain OR instability OR insufficiency OR subluxation) AND (pregnancy OR parturition OR 'ante natal' OR 'ante natally' OR prenatal* OR ‘pre natal’ OR ‘pre natally’ OR antenatal* OR birth OR childbirth OR perinatal* OR ‘peri natal’ OR ‘peri natally’ OR postpartum OR 'post partum' OR postnatal* OR ‘post natal’ OR ‘post natally’) AND (risk* OR predict* OR prognos* OR persist*) NOT (fibroid* OR endometriosis OR cyst* OR haemorrhage OR neoplasm OR cancer OR malignant OR 'pelvic inflammatory disease' OR salpingitis OR osteoporosis OR placenta OR placental OR ultrasound)) | 830 |

| **Initial searches (4 Dec 2014)** | Number of citations |
| --- | --- |
| PubMed | 1738 |
| CINAHL | 278 |
| MIDIRS | 184 |
| PsycINFO | 62 |
| Embase | 830 |
| **Following initial duplicate detection in Endnote (order of importing: PubMed, CINAHL, MIDIRS, PsycINFO, EMBASE)** |  |
| PubMed | 1730 |
| CINAHL | 98 |
| MIDIRS | 39 |
| PsycINFO | 21 |
| Embase | 495 |
| **TOTAL** | 2383 |

| **Database search update (15 April 2017)** | Number of citations |
| --- | --- |
| PubMed | 275 |
| CINAHL | 91 |
| MIDIRS | 40 |
| PsycINFO | 19 |
| Embase | 273 |
| **Following initial duplicate detection in Endnote (order of importing: PubMed, CINAHL, MIDIRS, PsycINFO, EMBASE)** |  |
| PubMed | 275 |
| CINAHL | 21 |
| MIDIRS | 20 |
| PsycINFO | 9 |
| Embase | 201 |
| **TOTAL** | 526 |

| **Trial registry (Date)** | **Filters** | **Search** | **Number of citations** |
| --- | --- | --- | --- |
| ClinicalTrials.gov (15 April 2017) | Observational studies only | (pelvic OR pelvis OR sacroiliac OR “sacro iliac” OR sacral OR sacrum OR “pubic symphysis” OR “symphysis pubis” OR symphyseal OR lumbopelvic OR lumbar OR back) AND (pain OR instability OR insufficiency OR subluxation) | 584 |

| **Database search update (3 August 2020)** | Number of citations |
| --- | --- |
| PubMed | 569 |
| CINAHL | 201 |
| MIDIRS | 50 |
| PsycINFO | 25 |
| Embase | 455 |
| **Following initial duplicate detection in Endnote (order of importing: PubMed, CINAHL, MIDIRS, PsycINFO, EMBASE)** |  |
| PubMed | 571 |
| CINAHL | 96 |
| MIDIRS | 0 |
| PsycINFO | 15 |
| Embase | 308 |
| **TOTAL** | 990 |

| **Trial registry (Date)** | **Filters** | **Search** | **Number of citations** |
| --- | --- | --- | --- |
| ClinicalTrials.gov (3 August 2020) | Observational studies only | (pelvic OR pelvis OR sacroiliac OR “sacro iliac” OR sacral OR sacrum OR “pubic symphysis” OR “symphysis pubis” OR symphyseal OR lumbopelvic OR lumbar OR back) AND (pain OR instability OR insufficiency OR subluxation) | 493 |
