# Supplementary material for: Risk factors for pregnancy-related pelvic girdle pain: a scoping review
Source: BMC Pregnancy Childbirth. 2020 Nov 27;20:739. doi: 10.1186/s12884-020-03442-5 (PMC7694360; doi:10.1186/s12884-020-03442-5)
Supplement: Supplementary file 2 — Additional file 2. Risk factors for PPGP examined in more than one study. [file 12884_2020_3442_MOESM2_ESM.docx]

**Additional file 2: Risk factors for PPGP examined in more than one study**

Table 7: Physical risk factors for PPGP examined in more than one study

| **Factor** | **Comparisons** | **Study** | **Participants (all or subgroup)** | **Trimester follow up** | **Outcome** | **No of participants** | **Unadjusted OR unless stated otherwise** | **Adjusted OR unless stated otherwise** |
| --- | --- | --- | --- | --- | --- | --- | --- | --- |
| **Low back pain history** | Low back pain history (yes vs no) | Bjelland et al 2010 | All | 3^rd^ | Pelvic Girdle Syndrome^b^ | 75939 | 1.6^c^ [1.6-1.7]; p<0.0001 | 1.7 [1.6-1.8]^d^; p<0.001 |
|  |  |  |  | 3^rd^ | Severe Pelvic Girdle Syndrome^b^ | 75939 | 1.9^c^ [1.7-2.1]; p<0.0001 | 2.0 [1.8-2.2]^d^; p<0.001 |
|  |  | Albert at al 2006 | All | 3^rd^ | PPGP | 2224 | 2.5^c^ [2-3.1]; p<0.0001 | 2.2^h^; p<0.001 |
|  |  |  |  | 3^rd^ | Pelvic Girdle Syndrome | 1880 | 3.1^c^ [2.1-4.4]; p<0.0001 | 2.3^h^; p<0.001 |
|  |  |  |  | 3^rd^ | Symphysiolysis | 1771 | 1.9^c^ [1.1-3.6]; p=0.03 | 1.8^h^; p=0.07 |
|  |  |  |  | 3^rd^ | One-sided sacroiliac syndrome | 1961 | 2.2^c^ [1.5-3.2]; p<0.0001 | 2.0^h^; p<0.01 |
|  |  |  |  | 3^rd^ | Double-sided sacroiliac syndrome | 1914 | 1.7^c^ [1.3-2.3]; p=0.0003 | 2.3^h^; p<0.001 |
|  |  | Pooled estimate^i^ | All | 3^rd^ | Pelvic Girdle Syndrome | 77809 | 2.2 [1.2-4.0]; I^2^=91%, Chi^2^=11.71, p=0.0006 | / |
| **History of low back pain not related to pregnancy^j^** | History of LBP not related to pregnancy (yes vs no) | Kovacs et al 2012 | All | 3^rd^ | PPGP | 1153 | 1.5^c^ [1.2-2.0]; p=0.0005 | / |
|  |  | Larsen et al 1999 | All | Any | PPGP | 1516 | / | 1.8 [1.2-2.6]^k^; p<0.01 |
| **Parity^j^** | Parity 1 vs 0 | Bjelland et al 2010 | All | 3^rd^ | Pelvic Girdle Syndrome^b^ | 62189 | 1.9^c^ [1.8-1.9]; p<0.0001 | 2.0 [1.9-2.1]^m^; p<0.001 |
|  |  |  |  | 3^rd^ | Severe Pelvic Girdle Syndrome^b^ | 62189 | 2.3^c^ [2.1-2.6]; p<0.0001 | 2.6 [2.3-2.9]^m^; p<0.001 |
|  |  | Endresen 1995 | All | Any | PPGP^b^ | 4055 | 1.9^c^ [1.6-2]; p<0.0001 | / |
|  |  |  |  | Any | PPGP^b^ no difficulties with housework | 2780 | 1.6^c^ [1.3-2]; p=0.0001 | / |
|  |  |  |  | Any | PPGP^b^ difficulties with housework to some degree | 3443 | 2.3^c^ [2.1-2.6]; p<0.0001 | / |
|  |  |  |  | Any | PPGP^b^ difficulties with housework to a large/high degree | 2786 | 2.7^c^ [2.1-3.5]; p<0.0001 | / |
|  | Parity 2 vs 0 | Bjelland et al 2010 | All | 3^rd^ | Pelvic Girdle Syndrome^b^ | 46296 | 2.3^c^ [2.2-2.4]; p<0.0001 | 2.6 [2.4-2.7]^m^; p<0.001 |
|  |  |  |  | 3^rd^ | Severe Pelvic Girdle Syndrome^b^ | 46296 | 3.2^c^ [2.9-3.7]; p<0.0001 | 3.8 [3.3-4.3]^m^; p<0.001 |
|  |  | Endresen 1995 | All | Any | PPGP^b^ | 3264 | 2.4^c^ [2.1-2.9]; p<0.0001 | / |
|  |  |  |  | Any | PPGP^b^ no difficulties with housework | 2257 | 2.2^c^ [1.6-2.9]; p<0.0001 | / |
|  |  |  |  | Any | PPGP^b^ difficulties with housework to some degree | 2758 | 2.3^c^ [2.1-2.6]; p<0.0001 | / |
|  |  |  |  | Any | PPGP^b^ difficulties with housework to a large/high degree | 2263 | 4.3^c^ [3.3-5.6]; p<0.0001 | / |
|  | Parity≥3 vs 0 | Bjelland et al 2010 | All | 3^rd^ | Pelvic Girdle Syndrome^b^ | 37684 | 2.3^c^ [2.1-2.6]; p<0.0001 | 2.6 [2.3-2.9]^m^; p<0.001 |
|  |  |  |  | 3^rd^ | Severe Pelvic Girdle Syndrome^b^ | 37684 | 3.3^c^ [2.7-4]; p<0.0001 | 3.6 [2.9-4.5]^m^; p<0.001 |
|  |  | Endresen 1995 | All | Any | PPGP^b^ | 2684 | 2.6^c^ [2.0-3.3]; p<0.0001 | / |
|  |  |  |  | Any | PPGP^b^ no difficulties with housework | 1937 | 2.7^c^ [1.8-4.2]; p<0.0001 | / |
|  |  |  |  | Any | PPGP^b^ difficulties with housework to some degree | 2321 | 2.3^c^ [2.1-2.6]; p<0.0001 | / |
|  |  |  |  | Any | PPGP^b^ difficulties with housework to a large/high degree | 1964 | 2.7^c^ [1.8-4.1]; p<0.0001 | / |
|  | Parity ≥1 vs 0 | Wergeland & Strand 1998 | All | Any | Disabling posterior pelvic pain (Posterior PPGP) | 3321 | 2.6^c^ [2-3.3]; p<0.0001 | / |
|  |  | Albert et al 2006 | All | 3^rd^ | PPGP | 2224 | 2.3^c^ [1.8-2.8]; p<0.0001 | 2.2^n^; p<0.001 |
|  |  |  |  | 3^rd^ | Pelvic Girdle Syndrome | 1880 | 3.7^c^ [2.4-5.5]; p<0.0001 | 3.5^n^; p<0.001 |
|  |  |  |  | 3^rd^ | Symphysiolysis | 1771 | 2.3^c^ [1.2-4.2]; p=0.008 | 2.7^n^; p<0.01 |
|  |  |  |  | 3^rd^ | One-sided sacroiliac syndrome | 1961 | 1.4^c^ [1-2]; p=0.07 | OR^c^ NS |
|  |  |  |  | 3^rd^ | Double-sided sacroiliac syndrome | 1914 | 2.5^c^ [1.8-3.4]; p<0.0001 | 2.4^n^; p<0.001 |
|  |  | Larsen et al 1999 | All | Any | PPGP | 1516 | 1.9^c^ [1.4-2.6]; p<0.0001 | / |
|  | Parity (continuous) | Larsen et al 1999 | All | Any | PPGP | 1516 | / | OR NS |
|  |  | Endresen 1995 | All | Any | PPGP^b^ | 2853 | / | β coefficient^n^ 0.2 (T-value 7.6); p<0.001 |
|  |  |  |  | Any | PPGP^b^ | 3062 | / | β coefficient^o^ 0.3 (T-value 9.8); p<0.001 |
|  |  |  |  | Any | PPGP^b^ + Often PLBP^b^ | 1116 | / | β coefficient^p^ 0.1 (T-value 3.0); p<0.01 |
|  |  |  |  | Any | PPGP Rarely/ never PLBP^b^ | 1737 | / | β coefficient^q^ 0.3 (T-value 8.2); p<0.001 |
| **Smoking^j^** | Smoking during pregnancy: occasional smoker (vs non-smoker) | Bjelland et al 2010 | All | 3^rd^ | Pelvic Girdle Syndrome^b^ | 71035 | 1.1^c^ [1.0-1.2]; p=0.1 | 1.0 [0.9-1.1]^s^ |
|  |  |  |  | 3^rd^ | Severe Pelvic Girdle Syndrome^b^ | 71035 | 1.5^c^ [1.2-1.9]; p=0.0007 | 1.2 [1.0-1.6]^s^ |
|  | Smoking during pregnancy: daily smoker (vs non-smoker) | Bjelland et al 2010 | All | 3^rd^ | Pelvic Girdle Syndrome^b^ | 73164 | 1.6^c^ [1.4-1.7]; p<0.0001 | 1.2 [1.1-1.3]^s^; p<0.001 |
|  |  |  |  | 3^rd^ | Severe Pelvic Girdle Syndrome^b^ | 73164 | 1.7^c^ [1.4-2.0]; p<0.0001 | 1.1 [0.9-1.3]^s^ |
|  | Past smoker | Meucci et al 2020 | All | Any | PPGP/pubic sympysis pain | 2689 | 1.0^c^ [0.7-1.6]; p=0.82 |  |
|  | Daily smoking (yes vs no) | Wergeland & Strand 1998 | All | Any | Disabling posterior pelvic pain (Posterior PPGP^b^) | 3311 | 1.7^c^ [1.3-2.1]; p<0.0001 | / |
|  | Smoking (yes vs no; unclear when) | Albert at al 2006 | All | 3^rd^ | PPGP | 2224 | 1.3^c^ [1-1.6]; p=0.03 | OR^t^ NS |
|  |  |  |  | 3^rd^ | Pelvic Girdle Syndrome | 1880 | 1.4^c^ [1.0-2.1]; p=0.05 | OR^t^ NS |
|  |  |  |  | 3^rd^ | Symphysiolysis | 1771 | 1.9^c^ [1.1-3.4]; p=0.03 | OR^t^ 2.2; p=0.05 |
|  |  |  |  | 3^rd^ | One-sided sacroiliac syndrome | 1961 | 1.4^c^ [0.9-2]; p=0.1 | OR^t^ NS |
|  |  |  |  | 3^rd^ | Double-sided sacroiliac syndrome | 1914 | 1.0^c^ [0.7-1.4]; p=0.8 | OR^t^ NS |
|  |  | Larsen et al 1999 | All | Any | PPGP | 1516 | 1.4^c^ [1.0-1.8]; p=0.04; Chi-squared test NS | / |
|  |  | Endresen 1995 | All | Any | PPGP^b^ | 2853 | / | β coefficient^u^ 0.07 (T-value 3.93); p<0.001 |
|  |  |  |  | Any | PPGP^b^ | 3062 | / | β coefficient^v^ 0.081 (T-value 4.41); p<0.001 |
|  |  |  |  | Any | PPGP^b^ Rarely/ never PLBP^b^ | 1737 | / | β coefficient^w^ 0.09 (T-value 4.34); p<0.001 |
|  |  | Meucci et al 2020 | All | Any | PPGP/pubic sympysis pain | 2689 | 1.0^c^ [0.7-1.6]; p=0.95 |  |
|  | Smoking quantity: 1-10/day (vs non-smoker) | Kovacs et al 2012 | All | 3^rd^ | PPGP | 1124 | 1.4^c^ [0.9-2.1]; p=0.2 | / |
|  | Smoking quantity: 11-20/day (vs non-smoker) | Kovacs et al 2012 | All | 3^rd^ | PPGP | 1017 | 2.0^c^ [0.4-9.5]; p=0.4 | / |
|  | Smoking quantity: >20/day (vs non-smoker) | Kovacs et al 2012 | All | 3^rd^ | PPGP | 1011 | 0.3^c^ [0.03-3.1]; p=0.3 | / |
| **BMI^j^** | Pre-pregnancy Body Mass Index (BMI) | Kovacs et al 2012 | All | 3^rd^ | PPGP | 1149 | Student t-test or Mann Whitney U test: p<0.01 | / |
|  | BMI (at 17 weeks gestation) 25-29 (vs <25) | Bjelland et al 2010 | All | 3^rd^ | Pelvic Girdle Syndrome^b^ | 63391 | 1.5^c^ [1.4-1.6]; p<0.0001 | 1.4 [1.3-1.5]^x^; p<0.001 |
|  |  |  |  | 3^rd^ | Severe Pelvic Girdle Syndrome^b^ | 63391 | 1.8^c^ [1.6-2.0]; p<0.0001 | 1.6 [1.4-1.8]^x^; p<0.001 |
|  | BMI (at 17 weeks gestation) ≥30 (vs <25) | Bjelland et al 2010 | All | 3^rd^ | Pelvic Girdle Syndrome^b^ | 50419 | 2.0^c^ [1.9-2.2]; p<0.0001 | 1.8 [1.7-1.9]^x^; p<0.001 |
|  |  |  |  | 3^rd^ | Severe Pelvic Girdle Syndrome^b^ | 50419 | 2.5^c^ [2.2-2.2]; p<0.0001 | 2.0 [1.7-2.3]^x^; p<0.001 |
|  | BMI >30 (yes vs no) (not stated when measured) | Albert at al 2006 | All | 3^rd^ | PPGP | 2224 | 1.3^c^ [0.8-2.1]; p=0.3 | / |
|  |  |  |  | 3^rd^ | Pelvic Girdle Syndrome | 1880 | 2.3^c^ [1.2-4.4]; p=0.009 | / |
|  |  |  |  | 3^rd^ | Symphysiolysis | 1771 | 0.5^c^ [0.07-3.9]; p=0.5 | / |
|  |  |  |  | 3^rd^ | One-sided sacroiliac syndrome | 1961 | 1.0^c^ [0.4-2.5]; p=1.0 | / |
|  |  |  |  | 3^rd^ | Double-sided sacroiliac syndrome | 1914 | 1.0^c^ [0.4-2.2]; p=1.0 | / |
|  | BMI (continuous; not stated when measured) | Endresen 1995 | All | Any | PPGP^b^ | 2853 | / | β coefficient^y^ 0.1 (T-value 2.2); p<0.05 |
|  |  |  |  | Any | PPGP^b^ | 3062 | / | β coefficient^z^ 0.2 (T-value 2.5); p<0.05 |
|  |  | Kovacs et al 2012 | All | 3^rd^ | PPGP | 1158 | / | β coefficient 0.05 [0.01-0.09]^a2^; p=0.01 |
| **Weight before pregnancy^j^** | Weight before pregnancy (continuous) | Albert et al 2006 | All | 3^rd^ | PPGP | 2224 | / | OR^c2^ NS |
|  |  |  |  | 3^rd^ | Pelvic Girdle Syndrome | 1880 | / | 1.03^c2^; p<0.05 |
|  |  |  |  | 3^rd^ | Symphysiolysis | 1771 | / | 1.04^c2^; p<0.05 |
|  |  |  |  | 3^rd^ | One-sided sacroiliac syndrome | 1961 | / | OR^c2^ NS |
|  |  |  |  | 3^rd^ | Double-sided sacroiliac syndrome | 1914 | / | OR^c2^ NS |
|  |  | Larsen et al 1999 | All | Any | PPGP | 1516 | OR NS | / |
|  |  | Kovacs et al 2012 | All | 3^rd^ | PPGP | 1149 | Student t-test or Mann Whitney U test: p<0.01 | / |
| **Maternal height^j^** | Maternal height (continuous) | Albert et al 2006 | All | 3^rd^ | PPGP | 2224 | / | OR^e2^ NS |
|  |  |  |  | 3^rd^ | Pelvic Girdle Syndrome | 1880 | / | OR^e2^ NS |
|  |  |  |  | 3^rd^ | Symphysiolysis | 1771 | / | OR^e2^ NS |
|  |  |  |  | 3^rd^ | One-sided sacroiliac syndrome | 1961 | / | OR^e2^ 1.05 p<0.05 |
|  |  |  |  | 3^rd^ | Double-sided sacroiliac syndrome | 1914 | / | OR^e2^ NS |
|  |  | Kovacs et al 2012 | All | 3^rd^ | PPGP | 1149 | Student t-test or Mann Whitney U test: p=0.64 | / |
| **Gestational diabetes^j^** |  | Meucci et al 2020 | All | Any | PPGP/pubic sympysis pain | 2689 | 1.3^c^ [0.7-2.3]; p=0.45 | / |
|  |  | Lebel et al 2010 | All | Any | Symphysiolysis | 80988 | 1.8 [1.0-3.2]; p=0.03 | / |
| ^c^Calculated from raw data (95% CI calculated using natural logarithm method (Altman et al 1991); ^d^Adjusted for Maternal age, Parity, BMI, educational level, emotional distress, physical demanding work, smoking in pregnancy, pre-pregnancy physical activity weekly; ^h^Adjusted for Trauma to the back, Salpingitis previous year, Multiparae, Weight before pregnancy, weight increase in pregnancy, smoking, height, social group 5, daily stress level, work satisfaction; ^i^Bjelland et al 2010 and Albert et al 2006: pooled raw data for Pelvic Girdle Syndrome (random effects) but unable to pool adjusted OR because no exact measure of variance reported in Albert et al 2006; ^j^Decision made not to pool the data because of significant heterogeneity including different times of follow up, adjusted and unadjusted effect measures, different sub-outcomes, insufficient data and/or differences in definition; ^k^Adjusted for uncomfortable working position, working in draft and cold, exercising regularly (once a week), pelvic pain in a previous pregnancy, previous lower abdominal pain while not pregnant, parity, weight, heavy workloads, age, smoking; ^m^Adjusted for Maternal age, BMI, educational level, previous low back pain, emotional distress, physical demanding work, smoking in pregnancy, pre-pregnancy physical activity weekly; ^n^Adjusted for History of low back pain, Trauma to the back, Salpingitis previous year, Weight before pregnancy, weight increase in pregnancy, smoking, height, social group 5, daily stress level, work satisfaction; ^o^Adjusted for low back pain, smoking, weight of newborn, work bending forward, woman's year of birth, BMI; ^p^Adjusted for smoking, weight of newborn, work bending forward, woman's year of birth, BMI, strain at work, economic independence twisting and bending ; ^q^Adjusted for frequent lifts 10-20kg, twisting and bending, strain at work; ^s^Adjusted for Maternal age, Parity, BMI, educational level, previous LBP, emotional distress, physically demanding work, pre-pregnancy physical activity weekly; ^t^Adjusted for History of low back pain, Trauma to the back, Salpingitis previous year, Multiparae, Weight before pregnancy, weight increase in pregnancy, height, social group 5, daily stress level, work satisfaction; ^u^Adjusted for low back pain, Parity, weight of newborn, work bending forward, woman's year of birth, BMI; ^v^Adjusted for parity, weight of newborn, work bending forward, woman's year of birth, BMI, strain at work, economic independence twisting and bending; ^w^Adjusted for parity, woman's year of birth, weight of newborn, permanently employed; ^x^Adjusted for Maternal age, Parity, educational level, previous low back pain, emotional distress, physical demanding work, smoking in pregnancy, pre-pregnancy physical activity weekly; ^y^Adjusted for low back pain, Parity, smoking, weight of newborn, work bending forward, woman's year of birth; ^z^Adjusted for parity, smoking, weight of newborn, work bending forward, woman's year of birth, strain at work, economic independence twisting and bending; ^a2^Adjusted for stage of pregnancy, depression (BDI-II score); ^c2^Adjusted for history of low back pain, Trauma to the back, Salpingitis previous year, Multiparae, weight increase in pregnancy, smoking, height, social group 5, daily stress level, work satisfaction; ^e2^Adjusted for history of low back pain, trauma to the back, salpingitis previous year, multiparae, weight before pregnancy, weight increase in pregnancy, smoking, social group 5, daily stress level, work satisfaction | | | | | | | | |

Table 8: Sociodemographic risk factors for PPGP examined in more than one study

| **Factor** | **Comparison** | **Study** | **Participants (all or subgroup)** | **Trimester follow up** | **Outcome** | **No of participants** | **Unadjusted OR** | **Adjusted OR** |
| --- | --- | --- | --- | --- | --- | --- | --- | --- |
| **Age (older)^e^** | Age <25 years (vs ≥35) | Bjelland et al 2010 | All | 3^rd^ | Pelvic Girdle Syndrome^b^ | 20767 | 1.1^c^ [1.02-1.2]; p=0.009 | 1.6 [1.4-1.7]^d^; p<0.001 |
|  |  |  |  | 3^rd^ | Severe Pelvic Girdle Syndrome^b^ | 20767 | 1.2^c^ [1-1.4]; p=0.03 | 1.7 [1.4-2.1]^d^; p<0.001 |
|  | Age 25-34 years (vs ≥35) | Bjelland et al 2010 | All | 3^rd^ | Pelvic Girdle Syndrome^b^ | 66214 | 1.8^c^ [1.7-1.9]; p<0.0001 | 1.3 [1.2-1.4]^d^; p<0.001 |
|  |  |  |  | 3^rd^ | Severe Pelvic Girdle Syndrome^b^ | 66214 | 1.1^c^ [0.9-1.2]; p=0.3 | 1.4 [1.2-1.6]^d^; p<0.001 |
|  | Age 20-29 years (vs <20) | Meucci et al 2018 | All | Any | PPGP/pubic sympysis pain | 2689 | 2.0^c^ [1.2-3.3]; p=0.0084 | RR 2.0^k^ (1.2-3.4); p=0.02 |
|  | Age ≥30 years (vs <20) | Meucci et al 2018 | All | Any | PPGP/pubic sympysis pain | 2689 | 3.6^c^ [2.2-6.0]; p=0.0001 | RR 2.1^k^ (1.2-3.6); p=0.02 |
|  | Age | Larsen et al 1999 | All | Any | PPGP | 1516 | OR NS | / |
|  |  | Kovacs et al 2012 | All | 3^rd^ | PPGP | 1149 | Student t-test or Mann Whitney U test: p=0.7 | / |
|  | Age <25 years (vs ≥30) | Wergeland & Strand 1998 | All | Any | Disabling posterior pelvic pain (Posterior PPGP^b^) | 2038 | 0.7^c^ [0.5-1.0]; p=0.04 | / |
|  | Age 25-29 years (vs ≥30) | Wergeland & Strand 1998 | All | Any | Disabling posterior pelvic pain (Posterior PPGP^b^) | 2511 | 0.9^c^ [0.7-1.2]; p=0.6 | / |
|  | Age ≤19 years (vs 20-24) | Endresen 1995 | All | Any | PPGP^b^ | 1813 | 0.8^c^ [0.6-1]; p=0.04 | / |
|  |  |  |  | Any | PPGP^b^ no difficulties with housework | 1203 | 0.9^c^ [0.6-1.4]; p=0.7 | / |
|  |  |  |  | Any | PPGP^b^ difficulties with housework to some degree | 1496 | 0.9^c^ [0.6-1.1]; p=0.3 | / |
|  |  |  |  | Any | PPGP^b^ difficulties with housework to large/high degree | 1194 | 0.5^c^ [0.3-0.8]; p=0.006 | / |
|  | Age 25-29 years (vs 20-24) | Endresen 1995 | All | Any | PPGP^b^ | 3354 | 1.0^c^ [0.9-1.1]; p=0.9 | / |
|  |  |  |  | Any | PPGP^b^ no cause difficulties with housework | 2174 | 0.9^c^ [0.7-1.1]; p=0.3 | / |
|  |  |  |  | Any | PPGP^b^ difficulties with housework to some degree | 2775 | 1.1^c^ [0.9-1.2]; p=0.5 | / |
|  |  |  |  | Any | PPGP^b^ difficulties with housework to large/high degree | 2193 | 0.9^c^ [0.7-1.2]; p=0.5 | / |
|  | Age 30-34 years (vs 20-24) | Endresen 1995 | All | Any | PPGP^b^ | 2629 | 0.9^c^ [0.8-1.1]; p=0.2 | / |
|  |  |  |  | Any | PPGP^b^ no difficulties with housework | 1725 | 0.8^c^ [0.6-1]; p=0.07 | / |
|  |  |  |  | Any | PPGP^b^ difficulties with housework to some degree | 2242 | 0.8^c^ [0.6-0.9]; p=0.005 | / |
|  |  |  |  | Any | PPGP^b^ difficulties with housework to large/high degree | 1772 | 1.1^c^ [0.9-1.4]; p=0.4 | / |
|  | Age 35-39 years (vs 20-24) | Endresen 1995 | All | Any | PPGP^b^ | 1831 | 0.9^c^ [0.7-1.1]; p=0.3 | / |
|  |  |  |  | Any | PPGP^b^ no difficulties with housework | 1206 | 1.0^c^ [0.6-1.5]; p=0.9 | / |
|  |  |  |  | Any | PPGP^b^ difficulties with housework to some degree | 1492 | 0.8^c^ [0.6-1.1]; p=0.1 | / |
|  |  |  |  | Any | PPGP^b^ difficulties with housework to large/high degree | 1215 | 1.1^c^ [0.7-1.6]; p=0.8 | / |
|  | Age ≥40 years (vs 20-24) | Endresen 1995 | All | Any | PPGP^b^ | 1520 | 0.8^c^ [0.4-1.5]; p=0.5 | / |
|  |  |  |  | Any | PPGP^b^ no difficulties with housework | 992 | 0.3^]c^ [0.04-2.0]; p=0.2 | / |
|  |  |  |  | Any | PPGP^b^ difficulties with housework to some degree | 1243 | 0.8^c^ [0.3-1.7]; p=0.5 | / |
|  |  |  |  | Any | PPGP^b^ difficulties with housework to large/high degree | 1001 | 1.3^c^ [0.5-3.5]; p=0.6 | / |
| **Educational level^e^** | Educational level: less than high school/ primary or secondary 1 (vs university level) | Kovacs et al 2012 | All | 3rd | PPGP | 706 | 1.6^c^ [1.1-2.2]; p=0.02 | / |
|  |  | Wergeland & Strand 1998 | All | Any | Disabling posterior pelvic pain (posterior PPGP^b^) | 1966 | 1.2^c^ [0.9-1.7]; p=0.2 | / |
|  | Educational level: high school/ secondary 2 (vs university level) | Kovacs et al 2012 | All | 3rd | PPGP | 946 | 1.3^c^ [1.0-1.8]; p=0.03 | / |
|  |  | Wergeland & Strand 1998 | All | Any | Disabling posterior pelvic pain (posterior PPGP^b^) | 2439 | 1.3^c^ [1.0-1.8]; p=0.04 | / |
|  | Educational level <12 years (vs ≥17) | Bjelland et al 2010 | All | 3rd | Pelvic Girdle Syndrome^b^ | 21397 | 4.5^c^ [4.2-4.9]; p<0.0001 | 1.3 [1.1-1.4]^h^; p<0.001 |
|  |  |  |  | 3rd | Severe Pelvic Girdle Syndrome^b^ | 21397 | 3.3^c^ [2.7-3.9]; p<0.0001 | 1.8 [1.4-2.2]^h^; p<0.001 |
|  | Educational level 12 years (vs ≥17) | Bjelland et al 2010 | All | 3rd | Pelvic Girdle Syndrome^b^ | 36351 | 3.8^c^ [3.6-4.1]; p<0.0001 | 1.2 [1.1-1.3]^h^; p<0.001 |
|  |  |  |  | 3rd | Severe Pelvic Girdle Syndrome^b^ | 36351 | 2.5^c^ [2.2-3.0]; p<0.0001 | 1.7 [1.4-2.0]^h^; p<0.001 |
|  | Educational level 13-16 years (vs ≥17) | Bjelland et al 2010 | All | 3rd | Pelvic Girdle Syndrome^b^ | 45379 | 3.1^c^ [2.9-3.3]; p<0.0001 | 1.1 [1.1-1.2]^h^; p<0.001 |
|  |  |  |  | 3rd | Severe Pelvic Girdle Syndrome^b^ | 45379 | 1.7^c^ [1.5-2]; p<0.0001 | 1.4 [1.2-1.6]^h^; p<0.001 |
| **Work satisfac-tion^e^** | Work satisfaction | Albert at al 2006 | All | 3rd | PPGP | 2224 | / | 0.9^j^; p<0.01 |
|  |  |  |  | 3rd | Pelvic Girdle Syndrome | 1880 | / | 0.9^j^; p<0.05 |
|  |  |  |  | 3rd | Symphysiolysis | 1771 | / | OR^j^ NS |
|  |  |  |  | 3rd | One-sided sacroiliac syndrome | 1961 | / | OR^j^ NS |
|  |  |  |  | 3rd | Double-sided sacroiliac syndrome | 1914 | / | 0.9^j^; p<0.01 |
|  |  | Larsen et al 1999 | All | Any | PPGP | 1516 | 0.6^c^ [0.3-1.2]; p=0.1 | / |
| ^c^Calculated from raw data (95% CI calculated using natural logarithm method (Altman et al 1991); ^d^Adjusted for maternal age, BMI, educational level, previous low back pain, emotional distress, physically demanding work, smoking in pregnancy, prepregancy physical activity; ^e^Decision made not to pool the data because of significant heterogeneity including different times of follow up, adjusted and unadjusted effect measures, different sub-outcomes, insufficient data and/or differences in definition; ^h^Adjusted for Maternal age, Parity, BMI, previous LBP, emotional distress, physical demanding work, smoking in pregnancy, pre-pregnancy physical activity weekly; ^j^Adjusted for History of LBP, Trauma to the back, Salpingitis previous year, Multiparae, Weight before pregnancy, weight increase in pregnancy, smoking, height, social group 5, daily stress level; ^k^Adjusted for smoking, diabetes, depression during pregnancy, number of pregnancies. | | | | | | | | |
