# Supplementary material for: Risk factors for pregnancy-related pelvic girdle pain: a scoping review
Source: BMC Pregnancy Childbirth. 2020 Nov 27;20:739. doi: 10.1186/s12884-020-03442-5 (PMC7694360; doi:10.1186/s12884-020-03442-5)
Supplement: Supplementary file 3 — Additional file 3. Risk factors for PPGP in the third trimester of pregnancy. [file 12884_2020_3442_MOESM3_ESM.docx]

**Additional file 3: Risk factors for PPGP in the third trimester of pregnancy**

Table 9: Physical risk factors for PPGP in the third trimester of pregnancy (examined in only one study)

| **Factor** | **Study** | **Participants (all or subgroup)** | **Outcome** | **No of participants** | **Unadjusted Odd Ratio unless stated otherwise** | **Adjusted Odds Ratio unless stated otherwise** |
| --- | --- | --- | --- | --- | --- | --- |
| **History of postpartum low back pain** | Kovacs et al 2012 | All | PPGP | 1164 | 2.0^b^ [1.4-2.8]; p=0.0002 | / |
| **Experiencing low back pain around the time when getting pregnant** | Kovacs et al 2012 | All | PPGP | 1164 | 1.3^b^ [0.9-1.8]; p=0.11 | / |
| **Low back pain in previous pregnancies** | Kovacs et al 2012 | All | PPGP | 1164 | 1.8^b^ [1.3-2.5]; p=0.0003 | / |
| **Physically demanding work (yes vs no)** | Bjelland et al 2010 | All | Pelvic Girdle Syndrome^e^ | 68872 | 1.6^b^ [1.6-1.7]; p<0.0001 | 1.4 [1.4-1.5]^f^; p<0.001 |
|  |  |  | Severe Pelvic Girdle Syndrome^e^ | 68872 | 1.9^b^ [1.8-2.1]; p<0.0001 | 1.5 [1.4-1.7]^f^; p<0.001 |
| **Exercise frequency 1-2 times per week during pregnancy vs <1 per week** | Gjestland et al 2013 | All | PPGP^e^ | 2013 | 0.8^b^ [0.6-0.9] | 0.9 [0.7-1.1]^i^ |
| **Exercise frequency ≥3 times per week during pregnancy vs <1 per week** | Gjestland et al 2013 | All | PPGP^e^ | 1575 | 0.8 [0.7-1.0] | 0.8 [0.6-1.0]^i^ |
| **Exercise frequency before pregnancy 1–3 times/month (vs never)** | Owe et al 2015 | Nulliparous | Pelvic Girdle Syndrome^e^ | 7309 | Risk Ratio (RR) 0.9 [0.8-1.1] | RR 1.0 [0.8-1.1]^j^; RR 1.0 [0.8-1.1]^k^ |
| **Exercise frequency before pregnancy 1–2 times/week (vs never)** | Owe et al 2015 | Nulliparous | Pelvic Girdle Syndrome^e^ | 12500 | RR 0.9 [0.8-1.0] | RR 1.0 [0.9-1.1]^j^; RR 1.0 [0.9-1.1]^k^ |
| **Exercise frequency before pregnancy 3–5 times/week (vs never)** | Owe et al 2015 | Nulliparous | Pelvic Girdle Syndrome^e^ | 17349 | RR 0.7 [0.7-0.8] | RR 0.9 [0.8-1.0]^j^; RR 0.8 [0.8-0.9]^k^ |
| **Exercise frequency before pregnancy ≥6 times/week (vs never)** | Owe et al 2015 | Nulliparous | Pelvic Girdle Syndrome^e^ | 10306 | RR 0.8 [0.7-0.9] | RR 1.0 [0.9-1.1]^j^; RR 1.0 [0.9-1.1]^k^ |
| **Exercise type: Brisk walking (vs never)** | Owe et al 2015 | Nulliparous | Pelvic Girdle Syndrome^e^ | 7880 | RR 0.9 [0.8-1.0] | RR 1.0 [0.8-1.1]^j^; RR 1.0 [0.8-1.1]^k^ |
| **Exercise type: Non-weight bearing** | Owe et al 2015 | Nulliparous | Pelvic Girdle Syndrome^e^ | 6641 | RR 0.8 [0.7-1.0] | RR 0.9 [0.8-1.1]^j^; RR 0.9 [0.8-1.0]^k^ |
| **Exercise type: Low-impact exercises** | Owe et al 2015 | Nulliparous | Pelvic Girdle Syndrome^e^ | 8869 | RR 0.8 [0.7-0.9] | RR 0.9 [0.8-1.1]^j^; RR 0.9 [0.8-1.1]^k^ |
| **Exercise type: High-impact exercises** | Owe et al 2015 | Nulliparous | Pelvic Girdle Syndrome^e^ | 12964 | RR 0.7 [0.6-0.8] | RR 0.9 [0.8-1.0]^j^; RR 0.8 [0.7-0.9]^k^ |
| **Exercise type: Horseback riding** | Owe et al 2015 | Nulliparous | Pelvic Girdle Syndrome^e^ | 6020 | RR 0.9 [0.8-1.0) | RR 1.0 [0.8-1.1]^j^; RR 1.0 [0.8-1.1]^k^ |
| **Exercise type: Mixed exercises** | Owe et al 2015 | Nulliparous | Pelvic Girdle Syndrome^e^ | 10610 | RR 0.9 [0.8-1.0] | RR 1.0 [0.9-1.1]^j^; RR 1.0 [0.9-1.1]^k^ |
| **Hours of exercise per week before pregnancy** | Kovacs et al 2012 | All | PPGP | 1149 | Student t-test or Mann Whitney U test: p=0.3 | / |
| **Hours of exercise per week during pregnancy** | Kovacs et al 2012 | All | PPGP | 1149 | Student t-test or Mann Whitney U test: p=0.2 | / |
| **Physical activity level: minimally active vs sedentary** | Kovacs et al 2012 | All | PPGP | 379 | 1.1^b^ [0.7-1.8]; p=0.6 | / |
| **Physical activity level: moderately active vs sedentary** | Kovacs et al 2012 | All | PPGP | 582 | 0.7^b^ [0.5-0.9]; p=0.02 | / |
| **Physical activity level: active vs sedentary** | Kovacs et al 2012 | All | PPGP | 492 | 0.7^b^ [0.5-1.1]; p=0.1 | / |
| **Physical activity level: very active vs sedentary** | Kovacs et al 2012 | All | PPGP | 452 | 0.9^b^ [0.6-1.3]; p=0.5 | / |
| **Pre-pregnancy physical activity: < 1 per week vs ≥3 per week** | Bjelland et al 2010 | All | Pelvic Girdle Syndrome^e^ | 41070 | 1.1^b^ [1.0-1.1]; p=0.01 | 1.0 [0.9-1.0]^m^ |
|  |  |  | Severe Pelvic Girdle Syndrome^e^ | 41070 | 1.1^b^ [1.0-1.1]; p=0.01 | 0.9 [0.8-1.0]^m^ |
| **Pre-pregnancy physical activity: 1-2 per week vs ≥3 per week** | Bjelland et al 2010 | All | Pelvic Girdle Syndrome^e^ | 53827 | 1.0^b^ [1.0-1.1]; p=0.7 | 1.0 [0.9-1.0]^m^ |
|  |  |  | Severe Pelvic Girdle Syndrome^e^ | 53827 | 0.9^b^ [0.8-1.0]; p=0.1 | 0.9 [0.8-1.0]^m^ |
| **Stage of pregnancy (weeks)** | Kovacs et al 2012 | All | PPGP | 1158 (1149) | β coefficient 0.07 [0.03-0.1]; p=0.001 | / |
|  |  | Have been pregnant before | PPGP | 394 | β coefficient 0.1 [0.04-0.2]; p=0.002 | / |
| **Lifetime duration of oral contraceptive pills: Combined oral contraceptive pills < 1 year vs never** | Bjelland et al 2013 | All | Pelvic Girdle Syndrome^e^ | 28480 | 1.0 [1.0-1.1] | 1.0 [1.0-1.1]^n^ |
| **Lifetime duration of oral contraceptive pills: Combined oral contraceptive pills 1-3 year vs never** | Bjelland et al 2013 | All | Pelvic Girdle Syndrome^e^ | 38195 | 1.0 [0.9-1.0]; p<0.05 | 1.0 [0.9-1.1]^n^ |
| **Lifetime duration of oral contraceptive pills: Combined oral contraceptive pills 4-6 year +vs never** | Bjelland et al 2013 | All | Pelvic Girdle Syndrome^e^ | 40770 | 0.9 [0.8-0.9]; p<0.001 | 1.0 [1.0-1.1]^n^ |
| **Lifetime duration of oral contraceptive pills: Combined oral contraceptive pills 7-9 year vs never** | Bjelland et al 2013 | All | Pelvic Girdle Syndrome^e^ | 38418 | 0.8 [0.7-0.8]; p<0.001 | 1.0 [0.9-1.0]^n^ p<0.05 |
| **Lifetime duration of oral contraceptive pills: Combined oral contraceptive pills ≥ 10 years (vs never)** | Bjelland et al 2013 | All | Pelvic Girdle Syndrome^e^ | 35606 | 0.8 [0.8-0.9]; p<0.001 | 1.0 [1.0-1.1]^n^ |
| **Lifetime duration of oral contraceptive pills: progestin-only contraceptive pills < 1 year vs never** | Bjelland et al 2013 | All | Pelvic Girdle Syndrome^e^ | 87236 | 1.3 [1.2-1.4]; p<0.001 | 1.1 [1.0-1.1]^n^ |
| **Lifetime duration of oral contraceptive pills: progestin-only contraceptive pills 1-3 year vs never** | Bjelland et al 2013 | All | Pelvic Girdle Syndrome^e^ | 84257 | 1.1 [1.0-1.2]; p<0.05 | 1.0 [0.9-1.1]^n^ |
| **Lifetime duration of oral contraceptive pills: progestin-only contraceptive pills 4-6 year vs never** | Bjelland et al 2013 | All | Pelvic Girdle Syndrome^e^ | 81352 | 1.1 [0.8-1.3] | 1.1 [0.8-1.4]^n^ |
| **Lifetime duration of oral contraceptive pills: progestin-only contraceptive pills 7-9 year vs never** | Bjelland et al 2013 | All | Pelvic Girdle Syndrome^e^ | 81044 | 1.2 [0.9-1.7] | 1.1 [0.8-1.6]^n^ |
| **Lifetime duration of oral contraceptive pills: progestin-only contraceptive pills ≥ 10 years vs never** | Bjelland et al 2013 | All | Pelvic Girdle Syndrome^e^ | 80984 | 1.3 [1.0-2.0] | 1.5 [1.0-2.2]^n^ p<0.05 |
| **Combined OCP in last year before pregnancy vs no hormonal contraception** | Bjelland et al 2013 | All | Pelvic Girdle Syndrome^e^ | 82042 | 0.8 [0.8-0.9]; p<0.001 | 1.0 [0.9-1.0]^n^ |
|  |  | Primiparous | Pelvic Girdle Syndrome^e^ | 42486 | 0.9 [0.8-0.9]; p<0.001 | 0.9 [0.8-0.9]^o^ p<0.001 |
|  |  | Multiparous | Pelvic Girdle Syndrome^e^ | 39556 | 1.1 [1.1-1.2]; p<0.001 | 1.1 [1.0-1.2]^o^ p<0.01 |
| **Progestin-only contraceptive pills in last year before pregnancy vs no hormonal contraception** | Bjelland et al 2013 | All | Pelvic Girdle Syndrome^e^ | 57282 | 1.1 [1.0-1.2]; p<0.05 | 1.0 [0.9-1.1]^n^ |
|  |  | Primiparous | Pelvic Girdle Syndrome^e^ | 22604 | 1.1 [1.0-1.4] | 1.2 [0.9-1.5]^o^ |
|  |  | Multiparous | Pelvic Girdle Syndrome^e^ | 34678 | 0.9 [0.8-1.0]; p<0.05 | 1.0 [0.9-1.1]^o^ |
| **Progestin injection in last year before pregnancy vs no hormonal contraception** | Bjelland et al 2013 | All | Pelvic Girdle Syndrome^e^ | 52724 | 1.1 [0.9-1.4] | 1.0 [0.8-1.3]^n^ |
|  |  | Primiparous | Pelvic Girdle Syndrome^e^ | 22057 | 1.5 [0.9-2.3] | 1.3 [0.8-2.0]^o^ |
|  |  | Multiparous | Pelvic Girdle Syndrome^e^ | 30667 | 0.9 [0.7-1.3] | 0.9 [0.6-1.2]^o^ |
| **Progestin intrauterine devices in last year before pregnancy vs no hormonal contraception** | Bjelland et al 2013 | All | Pelvic Girdle Syndrome^e^ | 56603 | 1.5 [1.3-1.6]; p<0.001 | 1.2 [1.1-1.3]^n^ p<0.001 |
|  |  | Primiparous | Pelvic Girdle Syndrome^e^ | 24146 | 1.3 [0.9-1.8] | 1.3 [0.9-1.9]^o^ |
|  |  | Multiparous | Pelvic Girdle Syndrome^e^ | 34457 | 1.2 [1.1-1.3]; p<0.001 | 1.2 [1.1-1.3]^o^ p<0.001 |
| **Combined oral contraceptive pill 4 months before pregnancy vs no hormonal contraception in last year** | Bjelland et al 2013 | All | Pelvic Girdle Syndrome^e^ | 68120 | 0.9 [0.8-0.9]; p<0.001 | 1.0 [0.9-1.1]^o^ |
| **Progestin-only contraceptive pill 4 months before pregnancy vs no hormonal contraception in last year** | Bjelland et al 2013 | All | Pelvic Girdle Syndrome^e^ | 54886 | 1.1 [1.0-1.2] | 1.0 [0.9-1.1]^o^ |
| **Cessation of oral contraceptives 4 months before pregnancy vs no hormonal contraception in last year** | Bjelland et al 2013 | All | Pelvic Girdle Syndrome^e^ | 68628 | 0.8 [0.8-0.9]; p<0.001 | 0.9 [0.9-1.0]^o^ |
| **Combined oral contraceptive pill at the time of being pregnant vs no hormonal contraception in last year** | Bjelland et al 2013 | All | Pelvic Girdle Syndrome^e^ | 53682 | 1.1 [1.0-1.3] | 1.2 [0.9-1.4]^o^ |
| **Progestin-only contraceptive pill at the time of being pregnant vs no hormonal contraception in last year** | Bjelland et al 2013 | All | Pelvic Girdle Syndrome^e^ | 52688 | 1.2 [1.0-1.6] | 1.0 [0.8-1.3]^o^ |
| **Cessation of oral contraceptives at the time of being pregnant vs no hormonal contraception in last year** | Bjelland et al 2013 | All | Pelvic Girdle Syndrome^e^ | 85264 | 0.9 [0.8-0.9]; p<0.001 | 1.0 [0.9-1.0]^o^ |
| **Weight increase during pregnancy** | Albert et al 2006 | All | PPGP | 2224 | / | OR^p^ NS |
|  |  |  | Pelvic Girdle Syndrome | 1880 | / | OR^p^ NS |
|  |  |  | Symphysiolysis | 1771 | / | OR^p^ NS |
|  |  |  | One-sided sacroiliac syndrome | 1961 | / | OR^p^ NS |
|  |  |  | Double-sided sacroiliac syndrome | 1914 | / | OR^p^ 1.1; p<0.05 |
| **Pain location: pubic symphysis vs no pain** | Robinson et al 2010 | All | PPGP; Disability | 268 | 17.7 [6.8-28.6] | 14.0 [3.7-24.1]^r^ p=0.007; 11.8 [2.3-21.2]^s^ p=0.03 |
|  |  |  | PPGP; Pain intensity | 268 | 42.2 [27.7-60.6] | 40.4 [24.4-56.5]^r^ p<0.001; 35.5 [19.7-51.1]^s^ p<0.001 |
| **Pain location: posterior pain only vs no pain** | Robinson et al 2010 | All | PPGP; Disability | 268 | 10.7 [6.2-15.3] | 4.8 [-0.2-9.6]^r^ p=0.007; 3.4 [-1.0-7.8]^s^ |
|  |  |  | PPGP; Pain intensity | 268 | 23.5 [16.6-30.3] | 15.3 [7.8-22.8]^r^ p<0.001; 11.8 [4.3-19.2]^s^ p<0.001 |
| **Pain location: posterior and pubic symphysis pain vs no pain** | Robinson et al 2010 | All | PPGP; Disability | 268 | 24.5 [15.6-33.5] | 11.8 [2.6-21.0]^r^ p=0.007; 8.4 [-0.07-17.0]^s^ |
|  |  |  | PPGP; Pain intensity | 268 | 40.5 [26.9-54] | 26.0 [11.6-44.0]^r^ p<0.001; 16.5 [1.8-31.1]^s^ p<0.001 |
| **≥1 previous instrumental birth** | Kovacs et al 2012 | All | PPGP | 1164 | 1.9^b^ [1.4-2.6]; p<0.0001 | / |
| **≥1 previous caesarean** | Kovacs et al 2012 | All | PPGP | 1164 | 1.0^b^ [0.6-1.5]; p=0.8 | / |
| **≥1 previous epidural anaesthesia** | Kovacs et al 2012 | All | PPGP | 1164 | 1.5^b^ [1.2-2]; p=0.004 | / |
| **Disability rating index in early pregnancy** | Robinson et al 2010c | All | PPGP; Disability | 268 | 0.6^b^ [0.5-0.7]; p<0.001 | 0.5 [0.3-0.6]^u^; p<0.001 |
| **Trauma to the back** | Albert et al 2006 | All | PPGP | 2224 | / | OR^v^ 2.8; p<0.001 |
|  |  |  | Pelvic Girdle Syndrome | 1880 | 3.4^b^ [2.1-5.4]; p<0.0001 | OR^v^ 3.5; p<0.001 |
|  |  |  | Symphysiolysis | 1771 | 0.7^b^ [0.3-1.9]; p=0.5 | OR^v^ NS |
|  |  |  | One-sided sacroiliac syndrome | 1961 | 2.4^b^ [1.4-4.2]; p=0.002 | OR^v^ 2.3; p<0.01 |
|  |  |  | Double-sided sacroiliac syndrome | 1914 | 2.5^b^ [1.4-3.7]; p<0.001 | OR^v^ 2.5; p<0.001 |
| **Years since last pregnancy** | Albert et al 2006 | All | PPGP | 2224 | / | Excluded from multivariable analysis because not significant in univariate analysis |
|  |  |  | Pelvic Girdle Syndrome | 1880 | / |  |
|  |  |  | Symphysiolysis | 1771 | / |  |
|  |  |  | One-sided sacroiliac syndrome | 1961 | / |  |
|  |  |  | Double-sided sacroiliac syndrome | 1914 | / |  |
| **Salpingitis previous year** | Albert et al 2006 | All | PPGP | 2224 | 1.5^b^ [0.97-2.4]; p=0.07 | OR^w^ NS |
|  |  |  | Pelvic Girdle Syndrome | 1880 | 1.5^b^ [0.7-3.2]; p=0.3 | OR^w^ NS |
|  |  |  | Symphysiolysis | 1771 | 1.1^b^ [0.3-4.5]; p=0.9 | OR^w^ NS |
|  |  |  | One-sided sacroiliac syndrome | 1961 | 2.3^b^ [1.2-4.4]; p=0.01 | OR^w^ 2; p=0.06 |
|  |  |  | Double-sided sacroiliac syndrome | 1914 | 1.3^b^ [0.6-2.6]; p=0.5 | OR^w^ NS |
| **Hormone induced pregnancy** | Albert et al 2006 | All | PPGP | 2224 | 0.6^b^ [0.3-1.04]; p=0.07 |  |
|  |  |  | Pelvic Girdle Syndrome | 1880 | 1.0^b^ [0.5-2.3]; p=0.9 |  |
|  |  |  | Symphysiolysis | 1771 | 0.4^b^ [0.06-3]; p=0.4 |  |
|  |  |  | One-sided sacroiliac syndrome | 1961 | 0.5^b^ [0.1-1.5]; p=0.2 |  |
|  |  |  | Double-sided sacroiliac syndrome | 1914 | 0.4^b^ [0.2-1.2]; p=0.1 |  |
| **Oral Contraceptive Pill** | Albert et al 2006 | All | PPGP | 2224 | 0.9^b^ [0.7-1.1]; p=0.4 |  |
|  |  |  | Pelvic Girdle Syndrome | 1880 | 0.7^b^ [0.4-1.1]; p=0.09 |  |
|  |  |  | Symphysiolysis | 1771 | 1.0^b^ [0.5-1.9]; p=0.9 |  |
|  |  |  | One-sided sacroiliac syndrome | 1961 | 1.3^b^ [0.9-1.9]; p=0.2 |  |
|  |  |  | Double-sided sacroiliac syndrome | 1914 | 0.7^b^ [0.5-1.1]; p=0.1 |  |
| **Number of previous pregnancies: 2 vs 1** | Kovacs et al 2012 | All | PPGP | 1081 | 1.3^b^ [1.0-1.7]; p=0.08 | / |
| **Number of previous pregnancies: 3 vs 1** | Kovacs et al 2012 | All | PPGP | 804 | 2.0^b^ [1.0-3.9]; p=0.05 | / |
| **Number of previous pregnancies: 4 vs 1** | Kovacs et al 2012 | All | PPGP | 770 | 2.0^b^ [0.5-7.4]; p=0.3 | / |
| **Number of previous pregnancies: 5 vs 1** | Kovacs et al 2012 | All | PPGP | 761 | 1.8^b^ [0.2-17.5]; p=0.6 | / |
| **Current weight (3rd trimester of pregnancy)** | Kovacs et al 2012 | All | PPGP | 1149 | Student t-test or Mann Whitney U test: p<0.01 | / |
| **Age of menarche <11 years (vs ≥14 years)** | Bjelland et al 2011 | All | Pelvic Girdle Syndrome^e^ | 74973 | 1.8 [1.6-2.0] | 1.5 [1.3-1.7]^x^; 1.4 [1.2-1.6]^y^ |
| **Age of menarche 11 years (vs ≥14 years)** | Bjelland et al 2011 | All | Pelvic Girdle Syndrome^e^ | 74973 | 1.5 [1.4-1.7] | 1.3 [1.2-1.5]^x^; 1.3 [1.2-1.4]^y^ |
| **Age of menarche 12 years (vs ≥14 years)** | Bjelland et al 2011 | All | Pelvic Girdle Syndrome^e^ | 74973 | 1.3 [1.2-1.4] | 1.2 [1.1-1.3]^x^; 1.2 [1.1-1.3]^y^ |
| **Age of menarche 13 years (vs ≥14 years)** | Bjelland et al 2011 | All | Pelvic Girdle Syndrome^e^ | 74973 | 1.2 [1.1-1.3] | 1.1 [1-1.2]^x^; 1.1 [1-1.2]^y^ |
| **Age of menarche 14 years (vs ≥14 years)** | Bjelland et al 2011 | All | Pelvic Girdle Syndrome^e^ | 74973 | 1.8 [1.6-2] | 1.5 [1.3-1.7]^x^; 1.4 [1.2-1.6]^y^ |
| **Nausea (only) in early pregnancy** | Chortatos et al 2015 | All | Pelvic Girdle Syndrome^e^ | 4020 | 2.1 [1.9-2.2] | 1.9 [1.8-2.1]^z^ |
| **Nausea and vomiting in early pregnancy** | Chortatos et al 2015 | All | Pelvic Girdle Syndrome^e^ | 3946 | 2.5 [2.3-2.7] | 2.3 [2.1-2.4]^z^ |
| ^f^Adjusted for Maternal age, Parity, BMI, educational level, previous LBP, emotional distress, smoking in pregnancy, pre-pregnancy physical activity weekly; ^i^Adjusted for maternal age, parity, education, marital status, smoking and pre-pregnancy body mass index; ^j^Adjusted for pre-pregnancy BMI, maternal age, education, history of low back pain and history of depression; ^k^Adjusted of pre-pregnancy BMI and maternal age; ^m^Adjusted for Maternal age, Parity, BMI, educational level, previous LBP, emotional distress, physically demanding work, smoking in pregnancy; ^n^Adjusted for maternal age, parity, educational level, BMI, age at menarche, other pain conditions and premenstrual depressive symptoms; ^o^Adjusted for maternal age, parity, educational level, BMI, age at menarche, other pain conditions and premenstrual depressive symptoms; ^p^Adjusted for History of LBP, Trauma to the back, Salpingitis previous year, Multiparae, Weight before pregnancy, smoking, height, social group 5, daily stress level, work satisfaction; ^u^Adjusted for pain locations, P4 test, sum of pain provocation tests, HSCL-25 in early pregnancy, age, parity, marital status, education, use of contraceptive pills, ASLR test, pre-pregnancy history of low back pain, work condition, number of pain sites, and mean Fear Avoidance Beliefs Questionnaire score in early pregnancy, pain intensity in early pregnancy**;** ^v^Adjusted for history of LBP, Salpingitis previous year, Multiparae, Weight before pregnancy, weight increase in pregnancy, smoking, height, social group 5, daily stress level, work satisfaction; ^w^Adjusted for History of LBP, Trauma to the back, Multiparae, Weight before pregnancy, weight increase in pregnancy, smoking, height, social group 5, daily stress level, work satisfaction; ^x^Adjusted for BMI; ^y^Adjusted for BMI, maternal age, parity, educational level, previous low back pain, physically demanding work and emotional distress; ^z^Adjusted for age, body mass index, smoking during pregnancy, parity, education, and gender. | | | | | | |

Table 10: Psychological risk factors for PPGP in the third trimester of pregnancy (examined in only one study)

| **Factors** | **Study** | **Participants (all or subgroup)** | **Outcome** | **No of participants** | **Unadjusted OR unless stated otherwise** | **Adjusted OR** |
| --- | --- | --- | --- | --- | --- | --- |
| **Depression: slightly (vs not)** | Kovacs et al 2012 | All | PPGP | 1030 | 2.0^b^ [1.5-2.6]; p<0.0001 | / |
| **Depression: moderately (vs not)** | Kovacs et al 2012 | All | PPGP | 749 | 2.0^b^ [1.3-3.5]; p=0.009 | / |
| **Depression: seriously (vs not)** | Kovacs et al 2012 | All | PPGP | 681 | 4.3^b^ [1.0-20.0]; p=0.06 | / |
| **Depression (BDI=II score)** | Kovacs et al 2012 | All | PPGP | 1158 | β coefficient 0.07 [0.04-0.1]; p<0.001 | / |
|  |  | Have been pregnant before | PPGP | 394 | β coefficient 0.09 [0.04-0.14]; p=0.001 | / |
| **Daily stress levels** | Albert et al 2006 | All | PPGP | 2224 | / | OR^f^ 1.1; p<0.01 |
|  |  |  | Pelvic Girdle Syndrome | 1880 | / | OR^f^ 1.2; p<0.001 |
|  |  |  | Symphysiolysis | 1771 | / | OR^f^ NS |
|  |  |  | One-sided sacroiliac syndrome | 1961 | / | OR^f^ 1.1; p<0.05 |
|  |  |  | Double-sided sacroiliac syndrome | 1914 | / | OR^f^ NS |
| **Anxiety: Traces of anxiety (vs normal)** | Kovacs et al 2012 | All | PPGP | 1019 | 2.0^b^ [1.4-3]; p=0.0003 | / |
| **Anxiety: Pathological anxiety (vs normal)** | Kovacs et al 2012 | All | PPGP | 907 | 2.4^b^ [1.2-4.5]; p=0.01 | / |
| **State Anxiety (STAI-S)** | Kovacs et al 2012 | All | PPGP | 1149 | Student t-test or Mann Whitney U test: p<0.01 | / |
| **Trait Anxiety (STAI-T)** | Kovacs et al 2012 | All | PPGP | 1149 | Student t-test or Mann Whitney U test: p<0.01 | / |
| **Anxiety (STAI score)** | Kovacs et al 2012 | All | PPGP | 1149 | Student t-test or Mann Whitney U test: p<0.01 | / |
| **Emotional distress: yes (≥2) (vs no (<2))** | Bjelland et al 2010 | All | Pelvic Girdle Syndrome^l^ | 74710 | 1.8^b^ [1.7-1.9]; p<0.0001 | 1.6 [1.5-1.8]^i^ |
|  |  |  | Severe Pelvic Girdle Syndrome^l^ | 41070 | 2.4^b^ [2.1-2.7]; p<0.0001 | 2.0 [1.8-2.3]^i^ |
| ^f^Adjusted for History of LBP, Trauma to the back, Salpingitis previous year, Multiparae, Weight before pregnancy, weight increase in pregnancy, smoking, height, social group 5, work satisfaction; ^i^Adjusted for Maternal age, Parity, BMI, educational level, previous LBP, physical demanding work, smoking in pregnancy, pre-pregnancy physical activity weekly; | | | | | | |

Table 11: Socio-demographic risk factors for PPGP in the third trimester of pregnancy (examined in only one study)

| **Factor** | **Study** | **Participants (all or subgroup)** | **Outcome** | **No of participants** | **Unadjusted OR** | **Adjusted OR** |
| --- | --- | --- | --- | --- | --- | --- |
| **Social group 5 (no vocational training or professional education education)** | Albert et al 2006 | All | PPGP | 2224 | 1.1^b^ [0.9-1.4]; p=0.3 | OR^c^ NS |
|  |  |  | Pelvic Girdle Syndrome | 1880 | 1.9^b^ [1.3-2.8]; p=0.0004 | OR^c^ NS |
|  |  |  | Symphysiolysis | 1771 | 0.9^b^ [0.4-1.8]; p=0.8 | OR^c^ NS |
|  |  |  | One-sided sacroiliac syndrome | 1961 | 0.8^b^ [0.5-1.2]; p=0.3 | OR^c^ 0.5; p<0.05 |
|  |  |  | Double-sided sacroiliac syndrome | 1914 | 1.0^b^ [0.7-1.4]; p=0.8 | OR^c^ NS |
| **Work status: currently working vs not working** | Kovacs et al 2012 | All | PPGP | 1139 | 0.8^b^ [0.6-1.0]; p=0.03 | / |
| ^b^Calculated from raw data (95% CI calculated using natural logarithm method (Altman et al 1991); ^c^Adjusted for History of LBP, Trauma to the back, Salpingitis previous year, Multiparae, Weight before pregnancy, weight increase in pregnancy, smoking, height, daily stress level, work satisfaction | | | | | | |
