# Supplementary material for: Risk factors for pregnancy-related pelvic girdle pain: a scoping review
Source: BMC Pregnancy Childbirth. 2020 Nov 27;20:739. doi: 10.1186/s12884-020-03442-5 (PMC7694360; doi:10.1186/s12884-020-03442-5)
Supplement: Supplementary file 4 — Additional file 4. Risk factors for PPGP in any trimester of pregnancy/trimester not stated. [file 12884_2020_3442_MOESM4_ESM.docx]

**Additional file 4: Risk factors for PPGP in any trimester of pregnancy/trimester not stated**

Table 12: Physical risk factors for PPGP in any trimester of pregnancy (examined in only one study)

| **Factors** | **Study** | **Participants (all or subgroup)** | **Outcome** | **No of participants** | **Unadjusted OR** | **Adjusted OR unless stated otherwise** |
| --- | --- | --- | --- | --- | --- | --- |
| **Low back pain during pregnancy** | Endresen 1995 | All | PPGP^b^ | 2853 | / | β coefficient^c^ 0.514 (T-value 19.6); p<0.001 |
| **Symptom-giving pelvic girdle relaxation in mother or sister** | Larsen et al 1999 | All | PPGP | 1516 | 2.1^g^ [1.2-3.6]; p=0.01 | / |
| **Regular exercise (once a week)** | Larsen et al 1999 | All | PPGP | 1516 | 0.6^g^ [0.5-0.8]; p=0.0019 | 0.6 [0.4-0.9]^i^; p<0.01 |
| **Diseases in the back, bones, or joints** | Larsen et al 1999 | All | PPGP | 1516 | 2.4^g^ [1.6-3.5]; p<0.0001 | / |
| **Suffering from lower abdominal pain** | Larsen et al 1999 | All | PPGP | 1516 | 3.1^g^ [2.1-4.5]; p<0.0001 | / |
| **Other diseases (other than diseases in the back, bones, or joints)** | Larsen et al 1999 | All | PPGP | 1516 | 1.6^g^ [1.0-2.8]; p=0.07 | / |
| **Previous lower abdominal pain (while not pregnant)** | Larsen et al 1999 | All | PPGP | 1516 | / | 3.1 [1.9-5.15]^j^; p<0.01 |
| **Lifting heavy loads at work (10-20kg)** | Wergeland & Strand 1998 | All | Disabling posterior pelvic pain (Posterior PPGP^b^) | 3284 | 1.3^g^ [1.0-1.7]; p=0.04 | / |
| **Heavy loads to carry (>10kg)** | Larsen et al 1999 | All | PPGP | 1516 | 1.9^g^ [1.4-2.6]; p<0.0001; Chi-squared p<0.01 | / |
| **Strain at work (not clearly defined)** | Endresen 1995 | All | PPGP^b^ | 3062 | / | β coefficient^k^ 0.045 (T-value 1.87); p>0.05 |
| **Work bending forward** | Endresen 1995 | All | PPGP^b^ | 3062 | / | β coefficient^k^ 0.05 (T-value 2.68); p<0.05 |
| **Twisting and bending** | Endresen 1995 | All | PPGP^b^ | 3062 | / | β coefficient^k^ 0.039 (T-value 2.02); p<0.05 |
| **Uncomfortable working positions** | Larsen et al 1999 | All | PPGP | 1516 | 2.7^g^ [2.0-3.7]; p<0.0001 | 1.7 [1.1-2.5]^l^; p<0.05 |
| **Long walking distance at work** | Larsen et al 1999 | All | PPGP | 1516 | 2.0^g^ [1.5-2.7]; p<0.0001 | / |
| **Stairs more than 10 steps at work** | Larsen et al 1999 | All | PPGP | 1516 | 1.1^g^ [0.8-1.4]; p=0.6 | / |
| **Working in draft and cold** | Larsen et al 1999 | All | PPGP | 1516 | 1.5^g^ [1.2-2.0]; p=0.003 | 2.1 [1.4-3.1]^n^; p=0.01 |
| **Working with chemicals** | Larsen et al 1999 | All | PPGP | 1516 | 1.1^g^ [0.7-1.6]; p=0.7 | / |
| **≥ 4 cups of coffee (per day)** | Wergeland & Strand 1998 | All | Disabling posterior pelvic pain (Posterior PPGP^b^) | 3286 | 1.8^g^ [1.3-2.4]; p=0.0001 | / |
| **Treatment of low back pain by doctor (vs untreated)** | Larsen et al 1999 | All | PPGP | 869 | 1.6^g^ [1.0-2.8]; p=0.07 | / |
| **Treatment of low back pain by chiropractor (vs untreated)** | Larsen et al 1999 | All | PPGP | 1009 | 0.8^g^ [0.6-1.1]; p=0.2 | / |
| **Treatment of low back pain by physiotherapist (vs untreated)** | Larsen et al 1999 | All | PPGP | 1163 | 0.8^g^ [0.6-1.1]; p=0.2 | / |
| **Untreated low back pain** | Larsen et al 1999 | All | PPGP | 1516 | 1.5^g^ [1.1-2.0]; p=0.01 | / |
| **Pelvic girdle pain in previous pregnancies** | Larsen et al 1999 | All | PPGP | 1516 | 13.0 [7.9-21.6]; p<0.0001 | 9.2 [4.6-18.1]^o^; p<0.01 |
| **2-4 Number of previous pregnancies (versus 1)** | Muecci et al 2018 | All | PPGP, pubic symphysis pain | 2688 | 1.3^g^ [0.98-1.8]; p=0.06 | RR^s^ 1.05 [0.8-1.5]; p=0.35 |
| **5 or more Number of previous pregnancies (versus 1)** | Muecci et al 2018 | All | PPGP, pubic symphysis pain | 2688 | 1.0^g^ [0.5-1.9]; p=0.96 | RR^s^ 0.6 [0.3-1.1]; p=0.35 |
| **Weight of newborn (in g)** | Endresen 1995 | All | PPGP^b^ | 2853 | / | β coefficient^p^ 1.2E-0.4 (T-value 3.47); p<0.001 |
|  |  | All | PPGP^b^ | 3062 | / | β coefficient^q^ 1.33E-0.4 (T-value 3.72); p<0.001 |
|  |  | All | PPGP^b^ + Rarely/ never PLBP^b^ | 1737 | / | β coefficient^r^ 1.72E-04 (T-value 4.32); p<0.001 |
| ^c^Adjusted for Parity, smoking, weight of newborn, work bending forward, woman's year of birth, BMI; ^g^Calculated from raw data (95% CI calculated using natural logarithm method (Altman et al 1991); ^i^Adjusted for uncomfortable working position, working in draft and cold, pelvic pain during previous pregnancy, previous low back pain while not pregnant, previous lower abdominal pain while not pregnant, parity, weight, heavy workloads, age, smoking; ^j^Adjusted for Uncomfortable working position, working in draft and cold, regular exercise (once a week), pelvic pain during previous pregnancy, previous low back pain while not pregnant, parity, weight, heavy workloads, age, smoking; ^k^Adjusted for parity, smoking, weight of newborn, work bending forward, woman's year of birth, BMI, economic independence twisting and bending; ^l^Adjusted for working in draft and cold, regular exercise (once a week), pelvic pain during previous pregnancy, previous low back pain while not pregnant, previous lower abdominal pain while not pregnant, parity, weight, heavy workloads, age, smoking; ^n^Adjusted for uncomfortable working positions, regular exercise (once a week), pelvic pain during previous pregnancy, previous low back pain while not pregnant, previous lower abdominal pain while not pregnant, parity, weight, heavy workloads, age, smoking; ^o^Adjusted for Uncomfortable working position, working in draft and cold, exercising regularly (once a week), previous low back pain while not pregnant, previous lower abdominal pain while not pregnant, parity, weight, heavy workloads, age, smoking; ^p^Adjusted for LBP, Parity, smoking, work bending forward, woman's year of birth, BMI; ^q^Adjusted for parity, smoking, work bending forward, woman's year of birth, BMI, strain at work, economic independence twisting and bending; ^r^Adjusted for parity, woman's year of birth, smoking, permanently employed; ^s^Adjusted for age, depression during pregnancy, smoking, diabetes. | | | | | | |

Table 13: Socio-demographic risk factors for PPGP in any trimester of pregnancy (examined in only one study)

| **Factor** | **Study** | **Participants (all or subgroup)** | **Outcome** | **No of participants** | **Unadjusted OR** | **Adjusted OR unless stated otherwise** |
| --- | --- | --- | --- | --- | --- | --- |
| **Woman's year of birth** | Endresen 1995 | All | PPGP^b^ | 2853 | / | β coefficient^c^ 0.012 (T-value 2.52); p<0.05 |
|  |  |  | PPGP^b^ | 3062 | / | β coefficient^b^ 0.024 (T-value 5.08); p<0.001 |
|  |  |  | PPGP^b^ + Rarely/ never PLBP^b^ | 1737 | / | β coefficient^c^ 0.017 (T-value 3.37); p<0.01 |
| **Partner's education level: primary or secondary 9-10 years (vs university)** | Wergeland & Strand 1998 | All | Disabling posterior pelvic pain (Posterior PPGP^b^) | 1822 | 1.4^g^ [1.1-1.9]; p=0.02 | / |
| **Partner's education level: secondary 11-12 years (vs university)** | Wergeland & Strand 1998 | All | Disabling posterior pelvic pain (Posterior PPGP^b^) | 2275 | 1.1^g^ [0.9-1.5]; p=0.4 | / |
| **Being in work** | Larsen et al 1999 | All | PPGP | 1516 | 1.5^g^ [1-2.3]; p=0.06 | / |
| **Monotonous work** | Larsen et al 1999 | All | PPGP | 1516 | 1.2^g^ [0.8-1.8]; p=0.4 | / |
| **Working part-time** | Larsen et al 1999 | All | PPGP | 1516 | 1.0^g^ [0.7-1.4]; p=1.0 | / |
| **Shiftwork** | Larsen et al 1999 | All | PPGP | 1516 | 0.8^g^ [0.5-1.2]; p=0.2 | / |
| **Fixed salary** | Larsen et al 1999 | All | PPGP | 1516 | 1.1^g^ [0.3-5.1]; p=0.9 | / |
| **Living in a house (yes vs no)** | Larsen et al 1999 | All | PPGP | 1516 | 1.0^g^ [0.7-1.3]; p=1 | / |
| **Having more than 3 rooms at home** | Larsen et al 1999 | All | PPGP | 1516 | 1.4^g^ [1.0-2.2]; p=0.08 | / |
| **Having a lift at home** | Larsen et al 1999 | All | PPGP | 1516 | 0.6^g^ [0.3-1.3]; p=0.2 | / |
| **Having stairs with more than 10 steps at home** | Larsen et al 1999 | All | PPGP | 1516 | 0.9^g^ [0.6-1.1]; p=0.2 | / |
| **Living with or married to partner** | Larsen et al 1999 | All | PPGP | 1516 | 0.6^g^ [0.4-1.0]; p=0.07 | / |
| **Children at home** | Larsen et al 1999 | All | PPGP | 1516 | 2.2^g^ [1.6-3.1]; p<0.0001 | / |
| **Doing more than 50% of the housework** | Larsen et al 1999 | All | PPGP | 1516 | 1.2^g^ [0.9-1.6]; p=0.3 | / |
| **Influence on breaks at work (yes vs no)** | Wergeland & Strand 1998 | All | Disabling posterior pelvic pain (Posterior PPGP^b^) | 3272 | 0.7^g^ [0.5-0.9]; p=0.002 | / |
| **Influence on work pace (yes vs no)** | Wergeland & Strand 1998 | All | Disabling posterior pelvic pain (Posterior PPGP^b^) | 3272 | 0.9^g^ [0.7-1.2]; p=0.6 | / |
| **Level of work pace control: No (vs high)** | Wergeland & Strand 1998 | All | Disabling posterior pelvic pain (Posterior PPGP^b^) | 3321 | / | 1.6 [1.0-2.4]^i^ |
| **Level of work pace control: low (vs high)** | Wergeland & Strand 1998 | All | Disabling posterior pelvic pain (Posterior PPGP^b^) | 3321 | / | 1.0 [0.7-1.4]^i^ |
| **Level of work pace control: medium (vs high)** | Wergeland & Strand 1998 | All | Disabling posterior pelvic pain (Posterior PPGP^b^) | 3321 | / | 1.1 [0.8-1.5]^i^ |
| **Externally paced work (yes vs no)** | Wergeland & Strand 1998 | All | Disabling posterior pelvic pain (Posterior PPGP^b^) | 3280 | 1.1^g^ [0.9-1.4]; p=0.4 | / |
| **Manual work (yes vs no)** | Wergeland & Strand 1998 | All | Disabling posterior pelvic pain (Posterior PPGP^b^) | 3273 | 1.1^g^ [0.9-1.4]; p=0.3 | / |
| **Influence on work content (yes vs no)** | Wergeland & Strand 1998 | All | Disabling posterior pelvic pain (Posterior PPGP^b^) | 3262 | 1.0^g^ [0.8-1.3]; p=0.8 | / |
| **Work with video display terminals (yes vs no)** | Wergeland & Strand 1998 | All | Disabling posterior pelvic pain (Posterior PPGP^b^) | 3187 | 0.8^g^ [0.6-1.1]; p=0.1 | / |
| **Weekly hours of paid work ≥35 (yes vs no)** | Wergeland & Strand 1998 | All | Disabling posterior pelvic pain (Posterior PPGP^b^) | 3168 | 0.8^g^ [0.6-1]; p=0.1 | / |
| **Weekly hours of paid work >40 (yes vs no)** | Wergeland & Strand 1998 | All | Disabling posterior pelvic pain (Posterior PPGP^b^) | 3168 | 0.7^g^ [0.4-1]; p=0.08 | / |
| **Economic dependence** | Endresen 1995 | All | PPGP^b^ | 3062 | / | β coefficient^j^ 0.052 (T-value 2.1); p<0.05 |
| **Permanently employed** | Endresen 1995 | All | PPGP^b^ + Rarely/ Never PLBP^b^ | 1737 | / | β coefficient^k^ 0.102 (T-value 2.05); p<0.05 |
| ^c^Adjusted for LBP, Parity, smoking, weight of newborn, work bending forward, BMI; ^g^Calculated from raw data (95% CI calculated using natural logarithm method (Altman et al 1991); ^i^Adjusted for age, parity, education, smoking, and manual work, low-back pain; ^h^Adjusted for parity, smoking, weight of newborn, work bending forward, woman's year of birth, BMI, strain at work, twisting and bending; ^j^Adjusted for parity, smoking, weight of newborn, work bending forward, woman's year of birth, BMI, strain at work, twisting and bending; ^k^Adjusted for parity, woman's year of birth, weight of newborn, smoking. | | | | | | |

Table 14: Psychological risk factors for PPGP in any trimester of pregnancy (examined in only one study)

| **Factors** | **Study** | **Participants (all or subgroup)** | **Outcome** | **No of participants** | **Unadjusted OR** | **Adjusted OR unless stated otherwise** |
| --- | --- | --- | --- | --- | --- | --- |
| **Depression during pregnancy** | Muecci et al 2020 | All | PPGP, pubic symphysis pain | 2853 | 2.0^g^ [1.1-3.6]; p=0.02 | RR^b^ 2.74 [1.38-5.44]; p=0.004 |
| ^a^ Calculated from raw data (95% CI calculated using natural logarithm method (Altman et al 1991); ^b^Adjusted for age, number of pregnancies, smoking, diabetes | | | | | | |
