# Supplementary material for: Risk factors for pregnancy-related pelvic girdle pain: a scoping review
Source: BMC Pregnancy Childbirth. 2020 Nov 27;20:739. doi: 10.1186/s12884-020-03442-5 (PMC7694360; doi:10.1186/s12884-020-03442-5)
Supplement: Supplementary file 5 — Additional file 5. Studies excluded at full text selection by reason for exclusion. [file 12884_2020_3442_MOESM5_ESM.docx]

**Additional file 5: Studies excluded at full text selection by reason for exclusion**

| Article not in English (n=19) | Carvalho et al 2016 |
| --- | --- |
|  | Ciaghi et al 2015 |
|  | van Vugt et al 2009 |
|  | Lochmuller et al 2005 |
|  | Alanen 1999 |
|  | Schoellner et al 2001 |
|  | Larsen et al 2000 |
|  | Palot et al 1995 |
|  | Kierkegaard 1992 |
|  | Kogstad 1988 |
|  | Kogstad 1990 |
|  | Krsnjavi et al 1988 |
|  | Bret et al 1959 |
|  | Mann et al 2008 |
|  | Kusumi et al 2009 |
|  | Ghaderi et al 2013 |
|  | Nacir et al 2008 |
|  | More often backache during the pregnancy: Clinical aspects, risks, treatment and prevention of atypical complaints. Gynakologie fur Hausarzte. 1998 (authors not listed) |
|  | Palot et al 1995 (b) |
| Intervention study (n=8) | Murphy et al 2009 |
|  | Weil et al 2008 |
|  | McIntyre et al 1996 |
|  | Ostgaard et al 1994 |
|  | Nikodem 2001 |
|  | Hasan 2007 |
|  | Brown et al 2004 |
|  | Peterson et al 2014 |
| Not original research (n=21) | Gorginzadeh et al 2016 |
|  | Pettigrew 2014 |
|  | Pettit et al 2014 |
|  | Young et al 2012 |
|  | Bigelow et al 2011 |
|  | Bailey 2009 |
|  | Stuge 2007 |
|  | Raheem 2003 |
|  | Cook et al 2001 |
|  | Rozenberg et al 1998 |
|  | Ostgaard 1996 |
|  | Paul et al 1994 |
|  | Taylor 2008 |
|  | Clinical digest. Nursing Standard. 2005 |
|  | Joy 2010 |
|  | Devine 1999 |
|  | Young 2002 |
|  | Sneag et al 2007 |
|  | Monier et al 2015 |
|  | Oates et al 2011 |
|  | More often backache during the pregnancy: Clinical aspects, risks, treatment and prevention of atypical complaints. Gynakologie fur Hausarzte. 1998. |
| Conference abstract (n=15) | Mena Iturriaga et al 2015 |
|  | Merry et al 2015 |
|  | Morino et al 2015 |
|  | Pumpure et al 2017 |
|  | Sila et al 2011 |
|  | Vinturache et al 2017 |
|  | Baron et al 2014 |
|  | Guzelkucnulluk et al 2014 |
|  | Dorheim et al 2013 |
|  | Gartland et al 2009 |
|  | Cubukcnullu et al 2009 |
|  | ABSTRACTS. Pediatrics. 2014 |
|  | Abstracts. Occupational Health Review. 2006 |
|  | BIRCWH and SCOR presentation abstracts. Journal of Women's Health, 2007 |
|  | Hamid et al 2012 |
| Follow-up at > 12 months postpartum (n=11) | Bergstrom et al 2014 |
|  | Bergstrom et al 2016 |
|  | Bjeland et al 2015 |
|  | Sjodahl et al 2013 |
|  | Rost et al 2006 |
|  | Albert et al 2001 |
|  | Brynhildsen et al 1998 |
|  | Ostgaard et al 1992 |
|  | To et al 2011 |
|  | Abitbol et al 1996 |
|  | Padua et al 2005 |
| Study protocol (n=3) | Bastiaanssen et al 2005 |
|  | Stomp-van den Berg et al 2007 |
|  | Brown et al 2006 |
| Case series/study (n=2) | Maigne et al 2012 |
|  | Khorshid et al 2004 |
| Pilot study (n=2) | Bewyer et al 2009 |
|  | Granath et al 2007 |
| Descriptive study (n=17) | Fakari et al 2018 |
|  | Gutke et al 2018 |
|  | Chang et al 2011 |
|  | Zasloff et al 2007 |
|  | Bo et al 2007 |
|  | Nilsson-Wikmar et al 2003 |
|  | Hansen et al 1999 |
|  | MacLennan et al 1997 |
|  | Bick et al 1997 |
|  | Robinson et al 2006 |
|  | Albert et al 2002 |
|  | Ostgaard et al 1996 |
|  | Wu et al 1992 |
|  | Rost et al 2004 |
|  | McEvoy et al 2001 |
|  | Woolhouse et al 2014 |
|  | Rodriguez et al 2004 |
| Back pain not specific to low back/pelvic girdle (n=20) | Limel et al 2016 |
|  | Lindgren et al 2014 |
|  | Mannion et al 2015 |
|  | Sabuncuoglu et al 2012 |
|  | Sabuncuoglu et al 2014 |
|  | Lindgren et al 2014 |
|  | Dumas et al 2010 |
|  | Cheng et al 2009 |
|  | To et al 2003 |
|  | Padua et al 2002 |
|  | Thompson et al 2002 |
|  | Bjorklund et al 2000 |
|  | Nilsson-Wikmar et al 1999 |
|  | Turgut et al 1998 |
|  | Paarlberg et al 2001 |
|  | Rodriguez et al 2001 |
|  | Smith et al 2008 |
|  | Russell et al 1993 |
|  | Hakansson et al 1993 |
|  | Ng et al 2017 |
| Symptoms may have started postpartum (n=29) | Tavares et al 2020 |
|  | Andersen et al 2015 |
|  | Beales et al 2016 |
|  | Chia et al 2016 |
|  | Cooklin et al 2015 |
|  | Olsson et al 2011 |
|  | Sperstad et al 2016 |
|  | Mota et al 2014 |
|  | Mukkannavar et al 2014 |
|  | Mukkannavar et al 2013 |
|  | Woolhouse et al 2012 |
|  | Stomp-van den Berg et al 2012 |
|  | Larsen et al 2013 |
|  | Biering et al 2011 |
|  | Gutke et al 2011 |
|  | Vollestad et al 2009 |
|  | Gutke et al 2008 |
|  | Patel et al 2007 |
|  | Van De Pol et al 2007 |
|  | Orlikowski et al 2006 |
|  | Juhl et al 2005 |
|  | Schytt et al 2005 |
|  | Stapleton et al 2002 |
|  | Breen et al 1994 |
|  | Gurel et al 1997 |
|  | MacArthur et al 1990 |
|  | Blomquist et al 2014 |
|  | Kainu et al 2010 |
|  | Vullo et al 1996 |
| Examined low back/pelvic girdle pain not related to pregnancy (n=8) | Bliddal et al 2016 |
|  | Wijnhoven et al 2006 |
|  | Lampe et al 2000 |
|  | Levangie 1999 |
|  | Sihvonen et al 1998 |
|  | Lotfi et al 2007 |
|  | Ziesat 1978 |
|  | Levangie 1998 |
| Focus on other outcomes (not PPGP) (n=27) | Kesikburun et al 2018 |
|  | Stafne et al 2019 |
|  | Drevin et al 2015 |
|  | Malmqvist et al 2015 |
|  | Morino et al 2016 |
|  | Dorheim et al 2012 |
|  | Dorheim et al 2013b |
|  | Perlen et al 2013 |
|  | Haakstad et al 2009 |
|  | Mogren 2006 |
|  | Sydsjo et al 2002 |
|  | Saugstad 1991 |
|  | Betz 1987 |
|  | Saraste 1986 |
|  | Knoepp et al 2013 |
|  | Stomp-van den Berg et al 2007 |
|  | di Cássia de Oliveira et al 2014 |
|  | MacArthur et al 1996 |
|  | Brown et al 2013 |
|  | Beaucage-Gauvreau et al 2011 |
|  | Wolf et al 2013 |
|  | Li et al 2014b |
|  | Yoo et al 2014 |
|  | Beaucage-Gauvreau et al 2012 |
|  | Mens et al 1996 |
|  | Kaerlev et al 2004 |
|  | Larsen et al 2006 |
| Examines other pelvic pain (e.g. visceral, perineal) (n=9) | Baron et al 2015 |
|  | Glowacka et al 2014 |
|  | Li et al 2014 |
|  | Hooker et al 2013 |
|  | Driul et al 2011 |
|  | Gaudet et al 2013 |
|  | Rosen 2016 |
|  | Paterson 2009 |
|  | Heisterberg 1993 |
| Reports development or evaluation of tests (n=10) | Fagevik Olsen et al 2013 |
|  | Fagevik Olsen et al 2009 |
|  | Ando et al 2009 |
|  | van de Pol et al 2006 |
|  | Bjorklund et al 1999 |
|  | Ostgaard et al 1994b |
|  | Kristiansson et al 1996 |
|  | Mens et al 2002 |
|  | Damen et al 2002 |
|  | Hansen et al 2005 |
| Looks at physical tests as factor (n=2) | Aldabe et al 2020 |
|  | Malmqvist et al 2018 |
| Non-adult population (n=1) | de Andrade et al 2018 |
| Prognostic factor studies (n=10) | Robinson et al 2010 |
|  | Bjelland et al 2013 |
|  | Bjelland et al 2013b |
|  | Bjelland et al 2013c |
|  | Mogren 2006 |
|  | Olsson et al 2012 |
|  | Mogren 2007 |
|  | Mogren 2008 |
|  | Mogren 2007b |
|  | Bjelland et al 2015 |
| Outcome: low back or lumbopelvic pain (n=21) | Fujimaki de Paula et al 2017 |
|  | Saxena et al 2019 |
|  | Sencan et al 2018 |
|  | Shijagurumayum Acharya et al 2019 |
|  | Venegas et al 2017 |
|  | Yetişgin et al 2019 |
|  | Elden et al 2016 |
|  | Gausel et al 2016 |
|  | Orvieta et al 1994 |
|  | Ostgaard et al 1991 |
|  | Kovacs et al 2012 |
|  | Wang et al 2004 |
|  | Malmqvist et al 2012 |
|  | Ostgaard et al 1991b |
|  | Mazicioglu et al 2006 |
|  | Berg et al 1988 |
|  | Endresen et al 1995 |
|  | Wergeland et al 1998 |
|  | Morino et al 2014 |
|  | Chang et al 2014 |
|  | Mohseni-Bandpei et al 2009 |
| Duplicate (n=1) | Fakari et al 2018 |
| Data on risk factors not shown in paper (n=1) | Weis et al 2018 |
